# Association Between Traumatic Brain Injury and Subsequent Cardiovascular Disease Among Post-9/11–Era Veterans

**DOI:** 10.1001/jamaneurol.2022.2682

**Published:** 2022-09-06

**Authors:** Ian J. Stewart, Megan E. Amuan, Chen-Pin Wang, Eamonn Kennedy, Kimbra Kenney, J. Kent Werner, Kathleen F. Carlson, David F. Tate, Terri K. Pogoda, Clara E. Dismuke-Greer, W. Shea Wright, Elisabeth A. Wilde, Mary Jo Pugh

**Affiliations:** 1 Department of Medicine, Uniformed Services University, Bethesda, Maryland; 2 Military Cardiovascular Outcomes Research Program, Bethesda, Maryland; 3 Informatics, Decision-Enhancement, and Analytic Sciences Center of Innovation, VA Salt Lake City Health Care System, Salt Lake City, Utah; 4 Division of Epidemiology, Department of Internal Medicine, University of Utah School of Medicine, Salt Lake City; 5 University of Texas Health San Antonio, Department of Population Health Sciences, San Antonio; 6 National Intrepid Center of Excellence, Walter Reed National Military Medical Center, Bethesda, Maryland; 7 Department of Neurology, Uniformed Services University, Bethesda, Maryland; 8 VA Portland Health Care System and Oregon Health and Science University, School of Public Health, Portland; 9 Department of Neurology, University of Utah School of Medicine, Salt Lake City; 10 VA Boston Healthcare System, Boston University School of Public Health, Boston, Massachusetts; 11 VA Palo Alto Healthcare System, Health Economics Resource Center, Palo Alto, California

## Abstract

**Importance:**

Traumatic brain injury (TBI) was common among US service members deployed to Iraq and Afghanistan. Although there is some evidence to suggest that TBI increases the risk of cardiovascular disease (CVD), prior reports were predominantly limited to cerebrovascular outcomes. The potential association of TBI with CVD has not been comprehensively examined in post-9/11–era veterans.

**Objective:**

To determine the association between TBI and subsequent CVD in post-9/11–era veterans.

**Design, Setting, and Participants:**

This was a retrospective cohort study conducted from October 1, 1999, to September 30, 2016. Participants were followed up until December 31, 2018. Included in the study were administrative data from the US Department of Veterans Affairs and the Department of Defense from the Long-term Impact of Military-Relevant Brain Injury Consortium–Chronic Effects of Neurotrauma Consortium. Participants were excluded if dates did not overlap with the study period. Data analysis was conducted between November 22, 2021, and June 28, 2022.

**Exposures:**

History of TBI as measured by diagnosis in health care records.

**Main Outcomes and Measures:**

Composite end point of CVD: coronary artery disease, stroke, peripheral artery disease, and cardiovascular death.

**Results:**

Of the 2 530 875 veterans from the consortium, after exclusions, a total of 1 559 928 veterans were included in the analysis. A total of 301 169 veterans (19.3%; median [IQR] age, 27 [23-34] years; 265 217 male participants [88.1]) with a TBI history and 1 258 759 veterans (80.7%; median [IQR] age, 29 [24-39] years; 1 012 159 male participants [80.4%]) without a TBI history were included for analysis. Participants were predominately young (1 058 054 [67.8%] <35 years at index date) and male (1 277 376 [81.9%]). Compared with participants without a history of TBI, diagnoses of mild TBI (hazard ratio [HR], 1.62; 95% CI, 1.58-1.66; *P* < .001), moderate to severe TBI (HR, 2.63; 95% CI, 2.51-2.76; *P* < .001), and penetrating TBI (HR, 4.60; 95% CI, 4.26-4.96; *P* < .001) were associated with CVD in adjusted models. In analyses of secondary outcomes, all severities of TBI were associated with the individual components of the composite outcome except penetrating TBI and CVD death.

**Conclusions and Relevance:**

Results of this cohort study suggest that US veterans with a TBI history were more likely to develop CVD compared with veterans without a TBI history. Given the relatively young age of the cohort, these results suggest that there may be an increased burden of CVD as these veterans age and develop other CVD risk factors. Future studies are needed to determine if the increased risk associated with TBI is modifiable.

## Key Points

**Question:**

What is the association of a traumatic brain injury (TBI) with the subsequent risk of cardiovascular disease in veterans of the recent conflicts in Iraq and Afghanistan?

**Findings:**

In this cohort study of 1 559 928 participants, TBI was associated with the development of a composite end point for cardiovascular disease (coronary artery disease, stroke, and peripheral artery disease). TBI was also associated with the individual components of this composite end point.

**Meaning:**

Traumatic brain injury is a potentially novel risk factor for cardiovascular disease in veterans.

## Teaser

This cohort study investigates the association of traumatic brain injury with subsequent risk of cardiovascular disease in veterans of the recent conflicts in Iraq and Afghanistan.

## Introduction

Since September 11, 2001, 4.5 million people have served in the US military,^1^ with their time in service defined by the long-running wars in Iraq and Afghanistan. As these conflicts finally come to an end, it is important to examine the potential long-term health effects of exposures these veterans encountered during their service. One potential exposure that could negatively affect long-term health is traumatic brain injury (TBI), which has been termed the signature injury of these conflicts.^2^ It is estimated that up to 20% of veterans who served in Iraq and Afghanistan sustained at least 1 TBI.^3^ TBI has been associated with a wide variety of adverse effects, including long-term disability,^4^ dementia,^5^ epilepsy,^6^ mental health conditions,^7^ and mortality.^8^

Cardiovascular disease (CVD) is caused by atherosclerosis and has several manifestations, including coronary artery disease (CAD), stroke, and peripheral artery disease (PAD). Although heart disease and stroke are the leading and fifth most common causes of death in the US, respectively,^9^ there are few studies that examine TBI as a risk factor for CVD. The best-studied CVD after TBI is stroke. Prior work has demonstrated associations between TBI and both hemorrhagic and ischemic stroke.^10,11,12^ However, these studies examined older, civilian populations and may not be generalizable to post-9/11–era veterans. Only 1 study^13^ has examined a broader definition of CVD. Nyam and colleagues^13^ examined a cohort of 16 211 individuals with TBI matched 1:2 with a control group from Taiwan’s National Health Insurance Research Database. They found that TBI was associated with a 72% increase in the subsequent rate of CAD. Despite a similar vascular pathogenesis as stroke and CAD, the potential relationship between TBI and PAD has not been examined.

Given the high prevalence of TBI in post-9/11–era veterans, the chronic nature of TBI sequelae, and prior data suggesting that TBI may be a risk factor for CVD, further analysis of long-term cardiovascular risks in this population is warranted. We hypothesized that TBI would be independently associated with CVD among post-9/11–era veterans (defined as service from fiscal year 2000 to 2016 regardless of deployment status) after adjustment for potentially confounding variables.

## Methods

### Participants

The research protocol was reviewed and approved by the University of Utah institutional review board (IRB) and was conducted in accordance with all applicable Federal regulations. The IRB granted a waiver for informed consent; the research involved no more than minimal risk, could not be carried out without the waiver, and the waiver did not affect the rights or welfare of the study participants. We used a variety of data sources including the US Department of Veterans Affairs (VA) and Department of Defense (DoD) Identity Repository (VADIR), the VA Corporate Data Warehouse (CDW), the DoD and VA Infrastructure for Clinical Intelligence (DaVINCI), the Theater Data Management Store (TMDS), the DoD Trauma Registry (DoDTR), and the National Death Index (NDI). Our cohort included participants from the Long-term Impact of Military-Relevant Brain Injury Consortium–Chronic Effects of Neurotrauma Consortium (LIMBIC-CENC) Phenotype study. To be included, participants had to have health care for at least 3 years in the DoD and, for those who entered the VA, 2 years of VA care. Veterans with an index date between October 1, 1999, and September 30, 2016, were included for analysis. LIMBIC-CENC included data before 9/11 in order to establish baseline rates. The competing risk was noncardiovascular death. Participants were censored at the date of their last health care system encounter or December 31, 2018 (whichever came first). To ensure adequate follow-up, those with an index date after October 1, 2016, were excluded. Veterans were also excluded from the analysis if they had only 1 outpatient diagnosis for CVD (unless it was a procedure or they subsequently had CVD death), had a CVD diagnosis before the index date, died before the index date (non-TBI cohort), were younger than 17 years at the index date, had an index date before their first DoD record, or had unclear TBI status. This study followed the Strengthening the Reporting of Observational Studies in Epidemiology (STROBE) reporting guidelines.

### Measures and Outcomes

Data on age, sex, education, component, rank, deployment history, and combat exposure were obtained from VADIR. Given previously described differences in cardiovascular health among different racial and ethnic groups,^14^ we obtained race and ethnicity variables from VADIR to account for potential health disparities. Veterans from the following race and ethnicity categories were included: Asian or Pacific Islander, Hispanic Black, Hispanic, Native American, non-Hispanic Black, non-Hispanic White, and unknown. Veterans who did not have a record of deployment could be classified as having combat exposure if they received a combat-zone tax exclusion or hostile fire or imminent-danger pay. Data on service branch were obtained from CDW. If this variable was missing from CDW, it was obtained from VADIR. Smoking status was determined by the method shown in eTable 1 in the Supplement. Data on mortality were obtained from VA vital status files and the NDI. Other covariates were defined by *International Classification of Diseases, Ninth and Tenth Revision, Clinical Modification (ICD-9-CM, ICD-10-CM)* diagnosis codes from DaVINCI, including hypertension (HTN), diabetes (DM), obesity, kidney disease, hyperlipidemia, obstructive sleep apnea (OSA), insomnia, depression, posttraumatic stress disorder (PTSD), anxiety, and substance use disorder (SUD) (eTable 2 in the Supplement). These were baseline covariates defined as present before the index date. Our definition of CVD was also based on *ICD-9-CM and ICD-10-CM* codes adapted from a prior published method^15^ (eTable 2 in the Supplement). TBI diagnosis was identified using a hierarchical approach prioritizing data from DoDTR and TMDS (Glasgow Coma Scale score, Abbreviated Injury Severity Score, and *ICD-9-CM *and* ICD-10-CM* codes), followed by self-reported loss of consciousness (mild, ≤30 minutes; moderate to severe, >30 minutes), alteration of consciousness or posttraumatic amnesia (mild, <24 hours; moderate to severe, ≥24 hours ^16^) identified in the VA comprehensive TBI evaluation, and *ICD-9-CM *and* ICD-10-CM *diagnosis codes from the 2012 Armed Forces Health Surveillance System algorithm.^17^

The primary outcome for our analysis was CVD, a composite of CAD, stroke, PAD, and CVD death (eTable 2 in the Supplement). To be diagnosed with CVD, veterans were required to have either 1 inpatient diagnosis or 2 outpatient diagnoses (at least 7 days apart) for any of these disorders. The secondary outcomes were the individual components of the composite outcome.

### Statistical Analysis

We used Fine-Gray competing risks models^18^ to estimate the association of TBI with subsequent CVD. For veterans with TBI, the index date was determined as the first date of a TBI diagnosis. If multiple TBI diagnoses were present, the most severe injury categorization was used. For veterans without TBI, index dates were simulated by drawing from the distribution of true index dates within each age bracket as previously described.^19^ In addition to bivariate analysis, we conducted 3 multivariable analyses in a nested fashion. To select variables for our models, we used a causal modeling approach and used directed acyclic graphs.^20^ In model 1, we adjusted for demographic variables: age, sex, race and ethnicity, education, service branch, component, rank, and deployment history. In model 2, we additionally adjusted for potential colliders: smoking, SUD, obesity, depression, anxiety, and insomnia. Lastly, in model 3, we additionally adjusted for potential mediators: PTSD, hyperlipidemia, HTN, kidney disease, DM, OSA, and insomnia. Our examination of secondary outcomes used this same methodology but considered the individual components of the primary end point as the outcomes. As an additional analysis to adjust for confounding, we incorporated inverse propensity score weighting (IPSW). In this analysis, inverse propensity scores associated with TBI status were incorporated as weights in the Fine-Gray competing risks model. The propensity scores associated with TBI were derived using multinomial regression modeling with predictors including demographic and baseline clinical characteristics. The propensity score model was evaluated in terms of covariate balance between TBI groups using standardized mean differences. To ensure doubly robust estimation of the effects of TBI on CVD incidence,^21^ all predictors in the propensity score model were included as predictors in the Fine-Gray competing risks model adjusted for the inverse propensity score weights associated with TBI status. To further examine whether the association of TBI with subsequent CVD varied over time, time-varying hazard ratios (HRs) were calculated. Data analysis was conducted between November 22, 2021, and June 28, 2022. All analyses were done on SAS Enterprise Guide, version 8.2 (SAS Institute). All tests were 2-tailed, and statistical significance was determined at an α level of .05.

## Results

**Figure 1.**
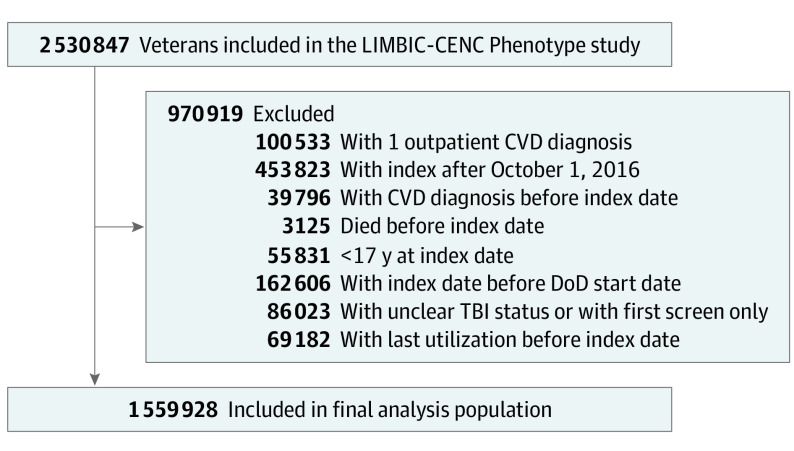
Consolidated Standards of Reporting Trials (CONSORT) Diagram Showing the Development of the Study Cohort CVD indicates cardiovascular disease; DoD, Department of Defense; LIMBIC-CENC, Long-term Impact of Military-Relevant Brain Injury Consortium-Chronic Effects of Neurotrauma Consortium.

The LIMBIC-CENC Phenotype study cohort is composed of 2 530 847 veterans. Of these, 970 919 were excluded, leaving 1 559 928 for analysis (Figure 1). A total of 301 169 veterans (19.3%; median [IQR] age, 27 [23-34] years; 265 217 male participants [88.1]; 35 898 female participants [11.9%]) with a TBI history and 1 258 759 veterans (80.7%; median [IQR] age, 29 [24-39] years; 1 012 159 male participants [80.4%]; 246 600 female participants [19.6%]) without a TBI history were included for analysis. Characteristics of the study cohort, stratified by TBI diagnosis, are shown in Table 1. Participants were predominately young (1 058 054 [67.8%] <35 years at index date) and male (1 277 376 [81.9%]). Veterans from the following race and ethnicity categories were included: (TBI history group) 23 789 Asian or Pacific Islander (7.9%), 1411 Hispanic Black (0.5%), 29 184 Hispanic (9.7%), 6083 Native American (2.0%), 42 996 non-Hispanic Black (14.3%), 194 841 non-Hispanic White (64.7%), and 2865 unknown (1.0%); (no TBI history group) 72 771 Asian or Pacific Islander (5.8%), 6997 Hispanic Black (0.6%), 120 438 Hispanic (9.6%), 20 669 Native American (1.6%), 224 996 non-Hispanic Black (17.9%), 789 136 non-Hispanic White (62.7%), and 23 752 unknown (1.9%). All variables examined were significantly different between the groups. Veterans with TBI were younger and more likely to be male than those without TBI history. A larger proportion of those without TBI history had post–high school education compared with those with TBI. Veterans with TBI were more likely to be active duty, in the Army or Marines, and have enlisted rank. They were also more likely to have deployed and have combat exposure. Compared with veterans without a TBI history, veterans with TBI were more likely to have a history of smoking (173 318 [57.6%] vs 497 203 [39.5%]; standardized mean difference [SMD], 0.37), SUD (47 616 [15.8%] vs 130 755 [10.4%]; SMD, 0.16), obesity (38 091 [12.7%] vs 157 393 [12.5%]; SMD, 0.004), OSA (20 890 [6.9%] vs 68 519 [5.4%]; SMD, 0.06), insomnia (55 677 [18.5%] vs 98 978 [7.9%]; SMD, 0.32), PTSD (82 543 [27.4%] vs 82 230 [6.5%]; SMD, 0.58), depression (74 704 [24.8%] vs 157 472 [12.5%]; SMD, 0.32), and anxiety (60 558 [20.1%] vs 119 947 [9.5%]; SMD, 0.30). Conversely, compared with veterans with a TBI history, the following diagnoses were more common in the group of veterans without TBI: hyperlipidemia (192 814 [15.3%] vs 37 598 [12.5%]; SMD, −0.08), kidney disease (5282 [0.4%] vs 1047 [0.4%]; SMD, −0.01), HTN (142 694 [11.3%] vs 32 220 [10.7%]; SMD, −0.02), and DM (25 481 [2.0%] vs 4388 [1.5%]; SMD, −0.04). Veterans with TBI had a mean (SD) follow-up of 7.3 (3.5) years, and veterans without TBI had a mean (SD) follow-up of 6.5 (3.2) years. The number of participants with each outcome, stratified by TBI severity, is shown in eTable 3 in the Supplement. Both the composite outcome and the individual components of the composite outcome were more common in the TBI group.

**Table 1.** Characteristics of the Study Cohort

Characteristic	No. (%)		Standardized mean difference^a^
	TBI history (n = 301 169)	No TBI history (n = 1 258 759)	
Age at index date, y			
17-24	105 157 (34.9)	354 645 (28.2)	0.15
25-34	123 563 (41.0)	474 689 (37.7)	0.07
35-44	48 244 (16.0)	238 370 (18.9)	−0.08
45-54	20 398 (6.8)	153 261 (12.2)	−0.19
55-64	3455 (1.2)	34 292 (2.7)	−0.11
≥65	352 (0.1)	3502 (0.3)	−0.04
Sex			0.21
Male	265 217 (88.1)	1 012 159 (80.4)	
Female	35 898 (11.9)	246 600 (19.6)	
Race and ethnicity			
Asian/Pacific Islander	23 789 (7.9)	72 771 (5.8)	0.08
Hispanic Black	1411 (0.5)	6997 (0.6)	−0.01
Hispanic	29 184 (9.7)	120 438 (9.6)	0.004
Native American	6083 (2.0)	20 669 (1.6)	0.03
Non-Hispanic			
Black	42 996 (14.3)	224 996 (17.9)	−0.10
White	194 841 (64.7)	789 136 (62.7)	0.04
Unknown	2865 (1.0)	23 752 (1.9)	−0.08
Education			
Less than high school	4642 (1.5)	17 576 (1.4)	0.01
High school	229 522 (76.2)	817 636 (65.0)	0.25
Some college	33 960 (11.3)	166 527 (13.2)	−0.06
College graduate	22 614 (7.5)	154 619 (12.3)	−0.16
Graduate school	8631 (2.9)	86 619 (6.9)	−0.19
Unknown	1794 (0.6)	15 782 (1.3)	−0.07
Service branch			
Army	176 663 (58.7)	531 511 (42.2)	0.33
Air Force	32 316 (10.7)	274 165 (21.8)	−0.30
Marines	54 624 (18.1)	180 255 (14.3)	0.10
Navy/Coast Guard	37 419 (12.4)	271 336 (21.6)	−0.25
Other	147 (0.1)	1492 (0.1)	−0.02
Component			
Guard	37 365 (12.4)	132 574 (10.5)	0.06
Reserve	102 389 (34.0)	486 975 (38.7)	−0.10
Active	161 415 (53.6)	639 240 (50.8)	0.06
Rank			
Officer	19 529 (6.5)	177 001 (14.1)	−0.25
Warrant	2521 (0.8)	15 859 (1.3)	−0.04
Enlisted	279 084 (92.7)	1 065 761 (84.7)	0.25
Deployment history			
+Combat and Deploy	235 195 (78.1)	783 481 (62.2)	0.35
+Combat/−Deploy	8001 (2.7)	68 697 (5.5)	−0.14
−Combat/+Deploy	12 413 (4.1)	66 365 (5.3)	−0.05
−Combat or Deploy	45 560 (15.1)	340 216 (27.0)	−0.29
Smoking history	173 318 (57.6)	497 203 (39.5)	0.37
Substance use disorder	47 616 (15.8)	130 755 (10.4)	0.16
Obesity	38 091 (12.7)	157 393 (12.5)	0.004
Obstructive sleep apnea	20 890 (6.9)	68 519 (5.4)	0.06
Insomnia	55 677 (18.5)	98 978 (7.9)	0.32
PTSD	82 543 (27.4)	82 230 (6.5)	0.58
Depression	74 704 (24.8)	157 472 (12.5)	0.32
Anxiety	60 558 (20.1)	119 947 (9.5)	0.30
Hyperlipidemia	37 598 (12.5)	192 814 (15.3)	−0.08
Kidney disease	1047 (0.4)	5282 (0.4)	−0.01
Hypertension	32 220 (10.7)	142 694 (11.3)	−0.02
Diabetes, types 1 and 2	4388 (1.5)	25 481 (2.0)	−0.04

Abbreviations: PTSD, posttraumatic stress disorder; TBI, traumatic brain injury.

^a^ Standardized mean difference for 2 groups is defined as the difference in means (for a continuous variable) or proportions (1 category at a time for categorical variables) divided by pooled SD.

**Figure 2.**
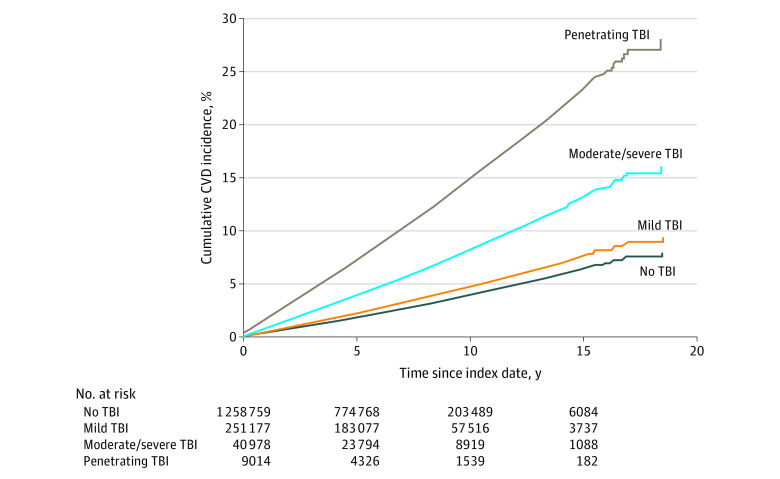
Cumulative Incidence Functions for the Composite Outcome Stratified by Traumatic Brain Injury (TBI) Severity CVD indicates cardiovascular disease.

**Figure 3.**
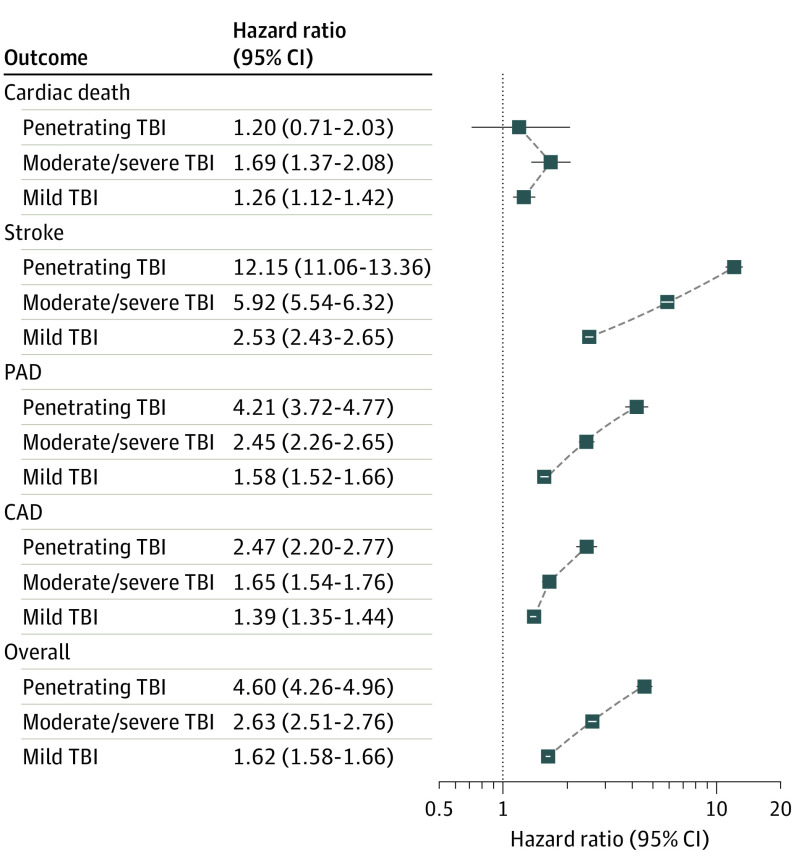
Hazard Ratio and 95% CIs for the Individual and Composite Outcomes in Fully Adjusted Models Stratified by Traumatic Brain Injury (TBI) CAD indicates coronary artery disease; PAD, peripheral artery disease.

The results from the models are shown in Table 2. For the bivariate analyses, mild TBI (HR, 1.18; 95% CI, 1.15-1.21; *P* < .001), moderate to severe TBI (HR, 2.10; 95% CI, 2.02-2.20; *P* < .001), and penetrating TBI (HR, 3.97; 95% CI, 3.70-4.25; *P* < .001) were significantly associated with CVD compared with veterans without TBI. For the multivariable analyses using model 3, compared with participants without history of TBI, diagnoses of mild TBI (HR, 1.62; 95% CI, 1.58-1.66; *P* < .001), moderate to severe TBI (HR, 2.63; 95% CI, 2.51-2.76; *P* < .001), and penetrating TBI (HR, 4.60; 95% CI, 4.26-4.96; *P* < .001) were associated with CVD. Figure 2 demonstrates this association graphically by showing the cumulative incidence functions. These findings remained significant in the multivariable models (Table 2). The results for the models that evaluated the individual components of the primary outcome (using the same covariates as in model 3) are shown in Figure 3. The results from the composite outcome are also included in this figure for reference. For the outcome of cardiovascular death, veterans with mild TBI (HR, 1.26; 95% CI, 1.12-1.42; *P* < .001) and moderate to severe TBI (HR, 1.69; 95% CI, 1.37-2.08; *P* < .001) were at significantly higher risk compared with veterans without TBI. Penetrating TBI did not appear to be associated with this outcome (HR, 1.20; 95% CI, 0.71-2.03; *P* = .50). All categories of TBI were significantly associated with stroke, PAD, and CAD. For each of these outcomes, there was the suggestion of a dose response whereby more severe TBI resulted in higher risk (Figure 3). The association with stroke was particularly prominent, with HRs ranging from 2.53 (95% CI, 2.43-2.65) for mild TBI to 12.15 (95% CI, 11.06-13.36) for penetrating TBI.

**Table 2.** Bivariate and Multivariable Competing Risk Models for the Outcome of Cardiovascular Disease

Model	Mild TBI^a^		Moderate to severe TBI^a^		Penetrating TBI^a^	
	HR (95% CI)	*P* value	HR (95% CI)	*P* value	HR (95% CI)	*P* value
Bivariate	1.18 (1.15-1.21)	<.001	2.10 (2.02-2.20)	<.001	3.97 (3.70-4.25)	<.001
Model 1^b^	1.74 (1.70-1.78)	<.001	2.89 (2.76-3.02)	<.001	5.03 (4.67-5.41)	<.001
Model 2^c^	1.57 (1.53-1.61)	<.001	2.54 (2.43-2.66)	<.001	4.49 (4.16-4.83)	<.001
Model 3^d^	1.62 (1.58-1.66)	<.001	2.63 (2.51-2.76)	<.001	4.60 (4.26-4.96)	<.001

Abbreviations: HR, hazard ratio; TBI, traumatic brain injury.

^a^ Compared with participants without TBI.

^b^ Adjusted for birth year, sex, race and ethnicity, education, service branch, component, rank, and deployment history.

^c^ Adjusted for birth year, sex, race and ethnicity, education, service branch, component, rank, deployment history, smoking history, substance use disorder, obesity, depression, anxiety, and insomnia.

^d^ Adjusted for birth year, sex, race and ethnicity, education, service branch, component, rank, deployment history, smoking history, substance use disorder, obesity, depression, anxiety, insomnia, posttraumatic stress disorder, hyperlipidemia, hypertension, kidney disease, diabetes, and obstructive sleep apnea.

In the IPSW-adjusted analysis of CVD, satisfactory covariate balance was achieved among the IPSW TBI groups (eTable 4 in the Supplement). In this analysis, mild TBI (HR, 1.66; 95% CI, 1.64-1.69; *P* < .001), moderate to severe TBI (HR, 2.74; 95% CI, 2.71-2.77; *P* < .001), and penetrating TBI (HR, 4.34; 95% CI, 4.30-4.39; *P* < .001) were all associated with increased CVD incidence compared with veterans without TBI. Testing the proportional hazards assumption suggested that the association of TBI with subsequent CVD incidence varied over time. The subsequent model allowing time-varying HRs (eTable 5 in the Supplement) found that the association of TBI was higher closer to the index date. HRs of CVD associated with mild TBI decreased from 3.67 (95% CI, 3.58-3.77; *P *< .001) at 3 months to 1.02 (95% CI, 1.00-1.03; *P *= .03) at 8 years. At years 9 (HR, 0.97; 95% CI, 0.96-0.99; *P *= .002) and 10 (HR, 0.94; 95% CI, 0.92-0.95; *P *< .001), this trend reversed and TBI was no longer associated with an increased risk of CVD. Similarly, the HRs of CVD associated with moderate to severe TBI decreased from 7.51 (95% CI, 7.33-7.69; *P *< .001) at 3 months to 1.08 (95% CI, 1.06-1.09; *P *< .001) at 8 years. At 9 years, moderate to severe TBI was not associated with CVD (HR, 1.01; 95% CI, 0.99-1.02; *P *= .28), and at 10 years, the trend reversed (HR, 0.95; 95% CI, 0.94-0.97; *P *< .001). Penetrating TBI was associated with an increased CVD incidence for all 10 years examined, with HRs ranging from 11.36 (95% CI, 11.10-11.63; *P* < .001) at 3 months to 1.81 (95% CI, 1.78-1.84; *P* < .001) at 10 years.

## Discussion

Results of this cohort study suggest that TBI was independently associated with CVD after adjustment for potentially confounding variables in a large cohort of post-9/11–era veterans. The association of TBI with subsequent CVD was not attenuated in multivariable models, suggesting that TBI may be accounting for risk that is independent from the other variables. When considering the components of the primary outcomes, all TBI categories increased the risk of stroke, CAD, and PAD after adjustment. Mild TBI and moderate to severe TBI were associated with an increased risk of CVD mortality. With the exception of CVD mortality, there was evidence for a dose response, whereby more severe TBI was associated with increased risk. Although the risk was highest shortly after injury, TBI remained significantly associated with CVD for years after the initial insult.

Only 1 study^13^ has examined the association of TBI with CVD broadly. Nyam et al^13^ examined the association of TBI with the subsequent incidence of major adverse cardiovascular and cerebrovascular events (MACCEs). This MACCE definition was inclusive of ischemic heart disease, stroke, and death identified using *ICD-9-CM* codes. The investigators found that TBI increased the incidence of subsequent MACCE with an HR of 2.77 (95% CI, 2.63-2.92). Consistent with our findings, the individual components of the MACCE outcome were also increased, with HRs of 1.72, 2.89, and 3.13 for ischemic heart disease, stroke, and death, respectively. Similar to the results of our analysis that minimized observed confounding using IPSW, the incidence of the MACCE outcomes shortly after injury were higher among the TBI groups compared with those without TBI. This study did not examine PAD, which we found to be associated with TBI after adjustment.

The best characterized cardiovascular outcome after TBI is stroke. The majority of this evidence is based on work from administrative records in Taiwan. Chen and colleagues^10^ studied 23 199 individuals with TBI compared with patients without TBI who were hospitalized or received ambulatory care from 2001 to 2003. They found that, although the risk of subsequent stroke was highest in the first 3 months after injury, the risk remained greater up to 5 years after injury. However, the majority of these events were hemorrhagic stroke. More recent work using this same database has extended the association of TBI with stroke to include mild TBI.^22^ TBI has also been shown to be a risk factor for subsequent stroke in other populations. Burke and colleagues^12^ examined emergency department and inpatient databases from 2005 to 2009 and compared patients with TBI (n = 436 630) to those with non-TBI trauma (n = 736 723). To avoid confounding by hemorrhagic stroke (which may be an immediate sequela of TBI), these investigators limited their analysis to ischemic stroke and found an association with TBI (HR, 1.31; 95% CI, 1.25-1.36) after adjustment.

The pathogenesis by which TBI is associated with subsequent CVD is unclear. It is possible that patients with TBI accumulate more traditional risk factors for CVD through time than patients without TBI. Although our analysis adjusted for these traditional risk factors at baseline, further work is required to elucidate the role these factors play after TBI. An association between TBI and CVD has also been observed in an animal model. Wang et al^23^ found that TBI leads to increased atherosclerosis in mice. This was despite similar cholesterol and blood pressure measures between TBI and sham mice. The authors found that mice with TBI had evidence of monocyte and neutrophil activation and increased markers of endothelial adhesiveness. In the context of our findings, this suggests that TBI results in an inflammatory state that may predispose individuals to atherosclerosis and subsequent CVD, independent of traditional pathways. Disruption of autonomic regulation has been noted to occur after TBI, which could serve as an additional mechanism.^24^ Multiple studies have demonstrated abnormalities in patients after concussion to include attenuated central regulation of sympathetic vasomotor tone,^25^ attenuated baroreflexes,^26,27^ and heart rate variability.^28^ Another potential pathway is through mental health diagnoses such as PTSD, as a large body of work has identified associations between PTSD and CVD,^29,30^ including among post-9/11 veterans.^31^ It has been postulated that this association could be explained by alterations in the hypothalamic-pituitary-adrenal axis, activation of the sympathetic nervous system, inflammation, and behavioral changes.^31^

### Strengths and Limitations

Our analysis had several strengths. First, it included a large cohort of post-9/11–era veterans. Second, our analysis included data from both the DoD and VA allowing for long follow-up intervals.

Our study also had several limitations. First, it was a retrospective analysis that was reliant on administrative data and was, therefore, subject to potential biases. Primary among these was that the date of TBI was the date that it was first diagnosed in the medical record, not necessarily when the TBI occurred. It is noteworthy, however, that our study used a robust definition and operationalization of TBI exposure using multiple data sources. Second, although we had complete data from the DoD and VA health care systems, we did not have data from private health care systems. This limitation is mitigated somewhat by the broadly consistent findings observed for CVD mortality, which was obtained from the NDI and was thus available for all veterans regardless of where they received their care. Third, a significant percentage of patients from the LIMBIC-CENC Phenotype study were excluded in this analysis (38%). The plurality of these exclusions were attributable to an index date after October 1, 2016, which did not allow sufficient time for follow up. Fourth, most covariates in our analysis were determined by *ICD-9-CM* and *ICD-10-CM* codes. Because ICD codes for cardiovascular risk factors are more specific than sensitive,^32^ our analysis may have underestimated the prevalence of these conditions at baseline. Lastly, the results from this relatively young, predominately White and male population may not be generalizable to other populations.

## Conclusions

In conclusion, results of this cohort study suggest that post-9/11–era veterans with a history of TBI were more likely to develop CVD than veterans without TBI. Furthermore, there was evidence to suggest a dose-response association, whereby more severe TBI was associated with higher CVD risk. Veterans with TBI in this study were relatively young, suggesting that there may be an increased burden of disease in the future as these patients age and develop traditional risk factors. Our results suggest that TBI was a significant risk factor for CVD. Further work is needed to determine how this risk can be modified to improve outcomes in post-9/11–era veterans.

## Notes

Supplement. **eTable 1.** Criteria for Determining Smoking Status **eTable 2. ** International Classification of Diseases, Ninth and Tenth Revision, Clinical Modification Codes Used to Determine Variables **eTable 3.** Outcomes Stratified by Traumatic Brain Injury Severity **eTable 4.** Weighted Baseline Characteristics of the Study Cohort After Inverse Propensity Score Weighting **eTable 5.** Time-Varying Risk in Inverse Propensity Score–Weighted Models
