# Supplementary material for: Association Between Traumatic Brain Injury and Subsequent Cardiovascular Disease Among Post-9/11–Era Veterans
Source: JAMA Neurol. 2022 Sep 6;79(11):1122–9. doi: 10.1001/jamaneurol.2022.2682 (PMC9449870; doi:10.1001/jamaneurol.2022.2682)
Supplement: Supplement. — eTable 1. Criteria for Determining Smoking Status eTable 2. International Classification of Diseases, Ninth and Tenth Revision, Clinical Modification Codes Used to Determine Variables eTable 3. Outcomes Stratified by Traumatic Brain Injury Severity eTable 4. Weighted Baseline Characteristics of the Study Cohort After Inverse Propensity Score Weighting eTable 5. Time-Varying Risk in Inverse Propensity Score–Weighted Models [file jamaneurol-e222682-s001.pdf]

Supplementary Online Content

Stewart IJ, Amuan ME, Wang CP, et al. Association between traumatic brain injury and subsequent cardiovascular disease among post-9/11–era veterans. *JAMA Neurol*. Published online September 6, 2022. doi:10.1001/jamaneurol.2022.2682

eTable 1. Criteria for Determining Smoking Status

eTable 2. *International Classification of Diseases, Ninth and Tenth Revision, Clinical Modification* Codes Used to Determine Variables

eTable 3. Outcomes Stratified by Traumatic Brain Injury Severity

eTable 4. Weighted Baseline Characteristics of the Study Cohort After Inverse Propensity Score Weighting

eTable 5. Time-Varying Risk in Inverse Propensity Score–Weighted Models

This supplementary material has been provided by the authors to give readers additional information about their work.

© 2022 Stewart IJ et al. *JAMA Neurology.*

eTable 1. Criteria for Determining Smoking Status

VA clinic stop codes	138, 707
International Classification of Diseases 9th and 10th Edition Clinical Modification	305.1, V1582, F17, Z87891, Z720
Prescriptions	Nicotine replacement<br>Varenicline<br>Bupropion (with the trade name Zyban
Positive clinical screen	Based on administrative records from both DaVINCI and VA data sources

eTable 2. *International Classification of Diseases, Ninth and Tenth Revision, Clinical Modification* Codes Used to Determine Variables

	**ICD9**	**ICD10**
Diabetes	UNCOMPLICATED<br>250.0X, 250.1X, 250.2X,250.3X<br><br>COMPLICATED<br>250.4X, 250.5X, 250.6X, 250.7X, 250.8X, 250.9X	UNCOMPLICATED<br>E100, E101, E109, E110, E111, E119, E120, E121, E129, E130, E131, E139, E140, E141, E149<br><br>COMPLICATED<br>E102, E103, E104, E105, E106, E107, E108, E112, E113, E114, E115, E116, E117, E118, E122, E123, E124, E125, E126, E127, E128, E132, E133, E134, E135, E136, E137, E138, E142, E143, E144, E145, E146,<br>E147, E148
Hypertension	UNCOMPLICATED<br>401.XX<br><br>COMPLICATED<br>402.XX, 403.XX, 404.XX, 405.XX	UNCOMPLICATED<br>I10<br><br>COMPLICATED<br>I11, I12, I13, I15
Hyperlipidemia	272.0X, 272.1X, 272.2X, 272.3X, 272.4X	E780, E781, E782, E783, E784, E785
Depression	296.2X, 296.3X, 311.XX	F32, F33 AND NOT F328
Anxiety	300.0X, 300.2X, 300.3X	F40, F41, F42
Kidney Disease	403.01, 403.11, 403.91, 404.02, 404.03, 404.12, 404.13, 404.92, 404.93, 585.XX, 586.XX, 588.0X, V42.0X, V45.1X, V56.XX	I120, I131, N18, N19, N250, Z490, Z491, Z492, Z940, Z992
Traumatic Brain Injury	Mild<br>80000, 80001, 80002, 80006, 80009, 80050, 80051, 80052, 80100, 80101, 80102, 80106, 80109, 80150, 80151, 80152,	Mild<br>F0781, S020XXA, S020XXB, S0210XA, S0210XB, S0291XA, S0291XB, S060X0A, S060X1A, S060X9A, S098XXA, S0990XA

© 2022 Stewart IJ et al. *JAMA Neurology.*

	80300, 80301, 80302, 80306, 80309, 80350, 80351, 80352, 80400, 80401, 80402, 80406, 80409, 80450, 80451, 80452, 8500, 8501, 85011, 3102, 95901, 850, 8505, 8509<br><br><u>Moderate</u><br>80003, 80010, 80011, 80012, 80013, 80016, 80019, 80020, 80021, 80022, 80023, 80026, 80029, 80030, 80032, 80031, 80033, 80036, 80039, 80040, 80041, 80042, 80043, 80046, 80049, 80053, 80056, 80059, 80103, 80110, 80111, 80112, 80113, 80116, 80119, 80120, 80121, 80122, 80123, 80126, 80129, 80130, 80131, 80132, 80133, 80136, 80139, 80140, 80141, 80142, 80143, 80146, 80149, 80153, 80156, 80159, 80303, 80310, 80311, 80312, 80313, 80316, 80319, 80320, 80321, 80322, 80323, 80326, 80329, 80330, 80331, 80332, 80333, 80336, 80339, 80340, 80341, 80342, 80343, 80346, 80349, 80353, 80356, 80359, 80403, 80410, 80411, 80412, 80413, 80416, 80419, 80420, 80421, 80422, 80423, 80426, 80429, 80430, 80431, 80432, 80433, 80436, 80439, 80440, 80441, 80442, 80443, 80446, 80449, 80453, 80456, 80459, 85012, 8502, 85100, 85101, 85102, 85103, 85106, 85109, 85120, 85121, 85122, 85123, 85126, 85129, 85140, 85141, 85142, 85143, 85146, 85149, 85160, 85161, 85162, 85163, 85166, 85169, 85180, 85181, 85182, 85183, 85186, 85189, 85200, 85201, 85202, 85203, 85206, 85209, 85220, 85221, 85222, 85223, 85226, 85229, 85240, 85241, 85242, 85243, 85246, 85249, 85300, 85301, 85302, 85303, 85306, 85309, 85401, 85402, 85403, 85406, 85409<br><br><u>Severe</u>	<u>Moderate</u><br>S020XXA, S020XXB, S0210XA, S0210XB, S0291XA, S0291XB, S060X0A, S060X2A, S060X3A, S060X4A, S061X0A, S061X1A, S061X2A, S061X3A, S061X4A, S061X9A, S06330A, S06331A, S06332A, S06333A, S06334A, S06339A, S06360A, S06361A, S06362A, S06363A, S06364A, S06369A, S06370A, S06371A, S06372A, S06373A, S06374A, S06379A, S06380A, S06381A, S06382A, S06383A, S06384A, S06389A, S064X0A, S064X1A, S064X2A, S064X3A, S064X4A, S064X9A, S065X0A, S065X1A, S065X2A, S065X3A, S065X4A, S065X9A, S066X0A, S066X1A, S066X2A, S066X3A, S066X4A, S066X9A, S06890A, S06891A, S06892A, S06893A, S06894A, S06899A, S069X0A, S069X1A, S069X2A, S069X3A, S069X4A, S069X9A<br><br><u>Severe</u><br>S020XXA, S020XXB, S0210XA, S0210XB, S0291XA, S0291XB, S060X5A, S060X6A, S061X5A, S061X6A, S061X7A, S061X8A, S06335A, S06336A, S06337A, S06338A, S06365A, S06366A, S06367A, S06368A, S06375A, S06376A, S06377A, S06378A, S06385A, S06386A, S06387A, S06388A, S064X5A, S064X6A, S064X7A, S064X8A, S065X5A, S065X6A, S065X7A, S065X8A, S066X5A, S066X6A, S066X7A, S066X8A, S06895A, S06896A, S06897A, S06898A, S069X5A, S069X6A, S069X7A, S069X8A<br><br><u>Penetrating</u><br>S0190XA, S020XXB, S0210XB, S0291XB, S06330A, S06331A, S06332A, S06333A, S06334A, S06335A, S06336A, S06337A, S06338A, S06339A, S06360A, S06361A, S06362A, S06363A, S06364A, S06365A, S06366A, S06367A, S06368A, S06369A, S06370A, S06371A, S06372A, S06373A, S06374A, S06375A, S06376A, S06377A, S06378A,

© 2022 Stewart IJ et al. *JAMA Neurology.*

	80004, 80005, 80014, 80015, 80024, 80025, 80034, 80035, 80044, 80045, 80054, 80055, 80104, 80105, 80114, 80115, 80124, 80125, 80134, 80135, 80144, 80145, 80154, 80155, 80304, 80305, 80314, 80315, 80324, 80325, 80334, 80335, 80344, 80345, 80354, 80355, 80404, 80405, 80414, 80415, 80424, 80425, 80434, 80435, 80444, 80445, 80454, 80455, 8503, 8504, 85104, 85105, 85124, 85125, 85144, 85145, 85164, 85165, 85184, 85185, 85204, 85205, 85224, 85225, 85244, 85245, 85304, 85305 85404, 85405<br><br><u>Penetrating</u><br>8006X, 8007X, 8008X, 8009X, 8016X, 8017X, 8018X, 8019X, 8036X, 8037X, 8038X, 8039X 8046X, 8047X, 8048X, 8049X, 8511X, 8513X, 8515X, 8517X, 8519X, 8521X, 8523X, 8525X, 8531X, 8541X))<br><br><u>Unclassified</u><br>9501, 9502, 9503, 9070, 85400<br><br><u>Personal History of Traumatic Brain Injury</u><br>V15.52	S06379A, S06380A, S06381A, S06382A, S06383A, S06384A, S06385A, S06386A, S06387A, S06388A, S06389A, S064X0A, S064X1A, S064X2A, S064X3A, S064X4A, S064X5A, S064X6A, S064X7A, S064X8A, S064X9A, S065X0A, S065X1A, S065X2A, S065X3A, S065X4A, S065X5A, S065X6A, S065X7A, S065X8A, S065X9A, S066X0A, S066X1A, S066X2A, S066X3A, S066X4A, S066X5A, S066X6A, S066X7A, S066X8A, S066X9A, S06890A, S06891A, S06892A, S06893A, S06894A, S06895A, S06896A, S06897A, S06898A, S06899A, S069X0A, S069X1A, S069X2A, S069X4A, S069X5A, S069X6A, S069X7A, S069X8A, S069X9A<br><br><u>Unclassified</u><br>S0402XA, S04039A, S04049A, S06890A, S069X9S<br><br><u>Personal History of Traumatic Brain Injury</u><br>Z87820
Insomnia	780.51, 780.52, 327.0X, 307.4X	F5101, F5102, F5103, F5104, F5105, F5109, G4700, G4701, G4709
Obstructive Sleep Apnea	327.23	G473
Obesity	278.0X	E40, E41, E42, E43, E44, E45, E46, R634, R64
Substance Use Disorder	<u>Opioid</u><br>304.0X, 304.7X, 305.5X, 965.0X, E8500, E9350<br><br><u>Alcohol</u><br>291.0X, 291.1X, 291.2X, 291.3X, 291.4X, 291.5X, 291.8X, 291.9X, 303.0X, 303.9X, 305.0X, 357.5X, 425.5X, 535.3X, 571.0X, 571.1X,571.2X, 571.3X, E8600, 980XX, V113X<br><br><u>Amphetamine</u>	<u>Opioid</u><br>F11, T400, T401, T402, T403<br><br><u>Alcohol</u><br>F10, G621, I426, K2920, K2921, K700, K7010, K709, T51, Z7141<br><br><u>Amphetamine</u><br>F15<br><br><u>Cannabis</u><br>F12, T407<br><br><u>Sedative</u>

© 2022 Stewart IJ et al. *JAMA Neurology.*

	304.4X, 305.7X<br><br><u>Cannabis</u><br>304.3X, 305.2X<br><br><u>Sedative</u><br>304.1X, 305.4X<br><br><u>Cocaine</u><br>304.2X, 305.6X, 968.5X, E9385<br><br><u>Hallucinogens</u><br>304.5X, 305.3X, 969.6X, E8541, E9396<br><br><u>Other Abuse</u><br>304.6X, 304.8X, 304.9X, 305.9X, 648.3X, V6542<br><br><u>Drug Induced Mental</u><br>292.0x, 292.11, 292.12, 292.2X, 292.81, 292.82, 292.83, 292.84, 292.85, 292.89, 292.9X	F13<br><br><u>Cocaine</u><br>F14, T405<br><br><u>Hallucinogens</u><br>F16, T408, T409<br><br><u>Other Abuse</u><br>F18, F19, O9932x<br><br><u>Drug Induced Mental</u><br>F11159, F11181, F11182, F11188, F11222, F11259, F11281, F11282, F11288, F11922, F11959, F11981, F11982, F11988, F12122, F12159,F12180, F12188, F12222, F12259, F12280, F12288, F12922, F12959, F12980, F12988, F13159, F13180, F13181, F13182, F13188, F13259,F13280, F13281, F13282, F13288, F13959, F13980, F13981, F13982, F13988, F14122, F14159, F14180, F14181, F14182, F14188, F14222, F14259, F14280, F14281, F14282, F14288, F14922, F14959, F14980, F14981, F14982, F14988, F15122, F15159, F15180, F15181, F15182, F15188, F15222, F15259, F15280, F15281, F15282, F15288, F15920, F15922, F15959, F15980, F15981, F15982, F15988, F16122, F16159, F16180, F16183, F16188, F16259, F16280, F16283, F16288, F16959, F16980, F16983, F16988, F17208, F17218, F17228, F17298, F18159, F18180, F18188, F18259, F18280, F18288, F18959, F18980, F18988, F19122, F19159, F19180, F19181, F19182, F19188, F19222, F19259, F19280, F19281, F19282, F19288, F19921, F19922, F19939, F1994, F19950, F19951, F19959, F1996, F1997, F19980, F19981, F19982, F19988, F1999
Post-traumatic Stress Disorder	309.81	F4310, F4312
Cardiovascular Disease	<u>Acute coronary heart disease</u><br>410.X-414.X (excluding 412)<br><br><u>Previous coronary heart disease</u>	<u>Acute coronary heart disease</u><br>I20X to I25X (excluding I252)<br><br><u>Previous coronary heart disease</u><br>I252

© 2022 Stewart IJ et al. *JAMA Neurology.*

	412<br><br>Cardiac arrest<br>427.5<br><br>Ischemic stroke, transient ischemic attack, or documented atherosclerotic cerebrovascular disease<br>434.X, 436.X, 437.1, 437.3, 438.X, 435.X<br><br>Peripheral arterial disease (PAD)<br>433.X, 441.X, 442.X, 444.X, 443.9, 440.21- 440.24<br><br>Coronary procedures<br>360.X, 361.X, 362.X<br><br>Peripheral arterial procedures<br>380.X, 381.X, 392.2-392.6, 392.8	Cardiac arrest<br>I46X, R96, R98<br><br>Ischemic stroke, transient ischemic attack, or documented atherosclerotic cerebrovascular disease<br>I63X, I64X, I66X, I670, I671, I693, I694, I698, G45X (except G453), G46X<br><br>Peripheral arterial disease (PAD)<br>I65X, I71X, I72X, I74X, I739, I7021, E1051, E1052, E1151, E1152, E1451, E1452<br><br>Coronary procedures<br>0210098, 0210099, 021009C, 021009F, 021009W, 02100A3, 02100A8, 02100A9, 02100AC, 02100AF, 02100AW, 02100J3, 02100J8, 02100J9, 02100JC, 02100JF, 02100JW, 02100K3, 02100K8, 02100K9, 02100KC, 02100KF, 02100KW, 02100Z3, 02100Z8, 02100Z9, 02100ZC, 02100ZF, 0210493, 0210498, 0210499, 021049C, 021049F, 021049W, 02104A3, 02104A8, 02104A9, 02104AC, 02104AF, 02104AW, 02104J3, 02104J8, 02104J9, 02104JC, 02104JF, 02104JW, 02104K3, 02104K8, 02104K9, 02104KC, 02104KF, 02104KW, 02104Z3, 02104Z8, 02104Z9, 02104ZC, 02104ZF, 0211098, 0211099, 021109C, 021109W, 02110A8, 02110A9, 02110AC, 02110AW, 02110J8, 02110J9, 02110JC, 02110JW, 02110K8, 02110K9, 02110KC, 02110KW, 02110Z8, 02110Z9, 02110ZC, 0211498, 0211499, 021149C, 021149W, 02114A8, 02114A9, 02114AC, 02114AW, 02114J8, 02114J9, 02114JC, 02114JW, 02114K8, 02114K9, 02114KC, 02114KW, 02114Z8, 02114Z9, 02114ZC, 021209C, 021209W, 02120AC, 02120AW, 02120JC, 02120JW, 02120KC, 02120KW, 02120ZC, 021249C, 021249W, 02124AC, 02124AW, 02124JC, 02124JW, 02124KC, 02124KW, 02124ZC, 021309C, 021309W, 02130AC, 02130AW,

© 2022 Stewart IJ et al. *JAMA Neurology.*

		02130JC, 02130JW, 02130KC, 02130KW, 02130ZC, 021349C, 021349W, 02134AC, 02134AW, 02134JC, 02134JW, 02134KC, 02134KW, 02134ZC, 021K0Z8, 021K0Z9, 021K0ZC, 021K0ZW, 021K4Z8, 021K4Z9, 021K4ZC, 021K4ZW, 021L0Z8, 021L0Z9, 021L0ZC, 021L4Z8, 021L4Z9, 021L4ZC, 02700ZZ, 02710ZZ, 02720ZZ, 02730ZZ, 02C00ZZ, 02C03ZZ, 02C04ZZ, 02C10ZZ, 02C13ZZ, 02C14ZZ, 02C20ZZ, 02C23ZZ, 02C24ZZ, 02C30ZZ, 02C33ZZ, 02C34ZZ, 3E07017, 3E070PZ, 3E07317, 3E073PZ, 021W09B, 021W09D, 021W0AB, 021W0AD, 021W0JB, 021W0JD, 021W0KB, 021W0KD, 021W0ZB, 021W0ZD, 021W49B, 021W49D, 021W4AB, 021W4AD, 021W4JB, 021W4JD, 021W4KB, 021W4KD, 021W4ZB, 021W4ZD, 02CP0ZZ, 02CP3ZZ, 02CP4ZZ, 02CQ0ZZ, 02CQ3ZZ, 02CQ4ZZ, 02CR0ZZ, 02CR3ZZ, 02CR4ZZ, 02CS0ZZ, 02CS3ZZ, 02CS4ZZ, 02CT0ZZ, 02CT3ZZ, 02CT4ZZ, 02CV0ZZ, 02CV3ZZ, 02CV4ZZ, 02CW0ZZ, 02CW3ZZ, 02CW4ZZ, 02HP0DZ, 02HP3DZ, 02HP4DZ, 02HQ0DZ, 02HQ3DZ, 02HQ4DZ, 02HR0DZ, 02HR3DZ, 02HR4DZ, 02HS02Z, 02HS0DZ, 02HS32Z, 02HS3DZ, 02HS42Z, 02HS4DZ, 02HT02Z, 02HT0DZ, 02HT32Z, 02HT3DZ, 02HT42Z, 02HT4DZ, 02HW02Z, 02HW0DZ, 02HW32Z, 02HW3DZ, 02HW42Z, 02HW4DZ, 031H09J, 031H0AJ, 031H0JJ, 031H0KJ, 031H0ZJ, 031J09K, 031J0AK, 031J0JK, 031J0KK, 031J0ZK, 031K09J, 031K0AJ, 031K0JJ, 031K0KJ, 031K0ZJ, 031L09K, 031L0AK, 031L0JK, 031L0KK, 031L0ZK, 031M09J, 031M0AJ, 031M0JJ, 031M0KJ, 031M0ZJ, 031N09K, 031N0AK, 031N0JK, 031N0KK, 031N0ZK, 031S09G, 031S0AG, 031S0JG, 031S0KG, 031S0ZG, 031T09G, 031T0AG, 031T0JG, 031T0KG, 031T0ZG, 039Y0ZZ, 039Y3ZZ, 039Y4ZZ, 03C00ZZ, 03C03ZZ, 03C04ZZ, 03C10ZZ,

© 2022 Stewart IJ et al. *JAMA Neurology.*

		03C13ZZ, 03C14ZZ, 03C20ZZ, 03C23ZZ, 03C24ZZ, 03C30ZZ, 03C33ZZ, 03C34ZZ, 03C40ZZ, 03C43ZZ, 03C44ZZ, 03C50ZZ, 03C53ZZ, 03C54ZZ, 03C60ZZ, 03C63ZZ, 03C64ZZ, 03C70ZZ, 03C73ZZ, 03C74ZZ, 03C80ZZ, 03C83ZZ, 03C84ZZ, 03C90ZZ, 03C93ZZ, 03C94ZZ, 03CA0ZZ, 03CA3ZZ, 03CA4ZZ, 03CB0ZZ, 03CB3ZZ, 03CB4ZZ, 03CC0ZZ, 03CC3ZZ, 03CC4ZZ, 03CD0ZZ, 03CD3ZZ, 03CD4ZZ, 03CF0ZZ, 03CF3ZZ, 03CF4ZZ, 03CG0ZZ, 03CG4ZZ, 03CH0ZZ, 03CH4ZZ, 03CJ0ZZ, 03CJ4ZZ, 03CK0ZZ, 03CK4ZZ, 03CL0ZZ, 03CL4ZZ, 03CM0ZZ, 03CM4ZZ, 03CN0ZZ, 03CN4ZZ, 03CP0ZZ, 03CP4ZZ, 03CQ0ZZ, 03CQ4ZZ, 03CR0ZZ, 03CR3ZZ, 03CR4ZZ, 03CS0ZZ, 03CS3ZZ, 03CS4ZZ, 03CT0ZZ, 03CT3ZZ, 03CT4ZZ, 03CU0ZZ, 03CU3ZZ, 03CU4ZZ, 03CV0ZZ, 03CV3ZZ, 03CV4ZZ, 03CY0ZZ, 03CY3ZZ, 03CY4ZZ, 03HY02Z, 03HY32Z, 03HY42Z, 0410090, 0410091, 0410092, 0410093, 0410094, 0410095, 0410096, 0410097, 0410098, 0410099, 041009B, 041009C, 041009D, 041009F, 041009G, 041009H, 041009J, 041009K, 041009Q, 041009R, 04100A0, 04100A1, 04100A2, 04100A3, 04100A4, 04100A5, 04100A6, 04100A7, 04100A8, 04100A9, 04100AB, 04100AC, 04100AD, 04100AF, 04100AG, 04100AH, 04100AJ, 04100AK, 04100AQ, 04100AR, 04100J0, 04100J1, 04100J2, 04100J3, 04100J4, 04100J5, 04100J6, 04100J7, 04100J8, 04100J9, 04100JB, 04100JC, 04100JD, 04100JF, 04100JG, 04100JH, 04100JJ, 04100JK, 04100JQ, 04100JR, 04100K0, 04100K1, 04100K2, 04100K3, 04100K4, 04100K5, 04100K6, 04100K7, 04100K8, 04100K9, 04100KB, 04100KC, 04100KD, 04100KF, 04100KG, 04100KH, 04100KJ, 04100KK, 04100KQ, 04100KR, 04100Z0, 04100Z1, 04100Z2, 04100Z3, 04100Z4, 04100Z5, 04100Z6, 04100Z7,

© 2022 Stewart IJ et al. *JAMA Neurology.*

		04100Z8, 04100Z9, 04100ZB, 04100ZC, 04100ZD, 04100ZF, 04100ZG, 04100ZH, 04100ZJ, 04100ZK, 04100ZQ, 04100ZR, 0410490, 0410491, 0410492, 0410493, 0410494, 0410495, 0410496, 0410497, 0410498, 0410499, 041049B, 041049C, 041049D, 041049F, 041049G, 041049H, 041049J, 041049K, 041049Q, 041049R, 04104A0, 04104A1, 04104A2, 04104A3, 04104A4, 04104A5, 04104A6, 04104A7, 04104A8, 04104A9, 04104AB, 04104AC, 04104AD, 04104AF, 04104AG, 04104AH, 04104AJ, 04104AK, 04104AQ, 04104AR, 04104J0, 04104J1, 04104J2, 04104J3, 04104J4, 04104J5, 04104J6, 04104J7, 04104J8, 04104J9, 04104JB, 04104JC, 04104JD, 04104JF, 04104JG, 04104JH, 04104JJ, 04104JK, 04104JQ, 04104JR, 04104K0, 04104K1, 04104K2, 04104K3, 04104K4, 04104K5, 04104K6, 04104K7, 04104K8, 04104K9, 04104KB, 04104KC, 04104KD, 04104KF, 04104KG, 04104KH, 04104KJ, 04104KK, 04104KQ, 04104KR, 04104Z0, 04104Z1, 04104Z2, 04104Z3, 04104Z4, 04104Z5, 04104Z6, 04104Z7, 04104Z8, 04104Z9, 04104ZB, 04104ZC, 04104ZD, 04104ZF, 04104ZG, 04104ZH, 04104ZJ, 04104ZK, 04104ZQ, 04104ZR, 0414093, 0414094, 0414095, 04140A3, 04140A4, 04140A5, 04140J3, 04140J4, 04140J5, 04140K3, 04140K4, 04140K5, 04140Z3, 04140Z4, 04140Z5, 0414493, 0414494, 0414495, 04144A3, 04144A4, 04144A5, 04144J3, 04144J4, 04144J5, 04144K3, 04144K4, 04144K5, 04144Z3, 04144Z4, 04144Z5, 041C090, 041C091, 041C092, 041C093, 041C094, 041C095, 041C096, 041C097, 041C098, 041C099, 041C09B, 041C09C, 041C09D, 041C09F, 041C09G, 041C09H, 041C09J, 041C09K, 041C09Q, 041C09R, 041C0A0, 041C0A1, 041C0A2,

		041C0A3, 041C0A4, 041C0A5,<br>041C0A6, 041C0A7, 041C0A8,<br>041C0A9, 041C0AB, 041C0AC,<br>041C0AD, 041C0AF, 041C0AG,<br>041C0AH, 041C0AJ, 041C0AK,<br>041C0AQ, 041C0AR, 041C0J0,<br>041C0J1, 041C0J2, 041C0J3,<br>041C0J4, 041C0J5, 041C0J6,<br>041C0J7, 041C0J8, 041C0J9,<br>041C0JB, 041C0JC, 041C0JD,<br>041C0JF, 041C0JG, 041C0JH,<br>041C0JJ, 041C0JK, 041C0JQ,<br>041C0JR, 041C0K0, 041C0K1,<br>041C0K2, 041C0K3, 041C0K4,<br>041C0K5, 041C0K6, 041C0K7,<br>041C0K8, 041C0K9, 041C0KB,<br>041C0KC, 041C0KD, 041C0KF,<br>041C0KG, 041C0KH, 041C0KJ,<br>041C0KK, 041C0KQ, 041C0KR,<br>041C0Z0, 041C0Z1, 041C0Z2,<br>041C0Z3, 041C0Z4, 041C0Z5,<br>041C0Z6, 041C0Z7, 041C0Z8,<br>041C0Z9, 041C0ZB, 041C0ZC,<br>041C0ZD, 041C0ZF, 041C0ZG,<br>041C0ZH, 041C0ZJ, 041C0ZK,<br>041C0ZQ, 041C0ZR, 041C490,<br>041C491, 041C492, 041C493,<br>041C494, 041C495, 041C496,<br>041C497, 041C498, 041C499,<br>041C49B, 041C49C, 041C49D,<br>041C49F, 041C49G, 041C49H,<br>041C49J, 041C49K, 041C49Q,<br>041C49R, 041C4A0, 041C4A1,<br>041C4A2, 041C4A3, 041C4A4,<br>041C4A5, 041C4A6, 041C4A7,<br>041C4A8, 041C4A9, 041C4AB,<br>041C4AC, 041C4AD, 041C4AF,<br>041C4AG, 041C4AH, 041C4AJ,<br>041C4AK, 041C4AQ, 041C4AR,<br>041C4J0, 041C4J1, 041C4J2,<br>041C4J3, 041C4J4, 041C4J5,<br>041C4J6, 041C4J7, 041C4J8,<br>041C4J9, 041C4JB, 041C4JC,<br>041C4JD, 041C4JF, 041C4JG,<br>041C4JH, 041C4JJ, 041C4JK,<br>041C4JQ, 041C4JR, 041C4K0,<br>041C4K1, 041C4K2, 041C4K3,<br>041C4K4, 041C4K5, 041C4K6,<br>041C4K7, 041C4K8, 041C4K9,<br>041C4KB, 041C4KC, 041C4KD,<br>041C4KF, 041C4KG, 041C4KH,<br>041C4KJ, 041C4KK, 041C4KQ,<br>041C4KR, 041C4Z0, 041C4Z1,<br>041C4Z2, 041C4Z3, 041C4Z4,<br>041C4Z5, 041C4Z6, 041C4Z7,<br>041C4Z8, 041C4Z9, 041C4ZB,

© 2022 Stewart IJ et al. *JAMA Neurology.*

		041C4ZC, 041C4ZD, 041C4ZF, 041C4ZG, 041C4ZH, 041C4ZJ, 041C4ZK, 041C4ZQ, 041C4ZR, 041D090, 041D091, 041D092, 041D093, 041D094, 041D095, 041D096, 041D097, 041D098, 041D099, 041D09B, 041D09C, 041D09D, 041D09F, 041D09G, 041D09H, 041D09J, 041D09K, 041D09Q, 041D09R, 041D0A0, 041D0A1, 041D0A2, 041D0A3, 041D0A4, 041D0A5, 041D0A6, 041D0A7, 041D0A8, 041D0A9, 041D0AB, 041D0AC, 041D0AD, 041D0AF, 041D0AG, 041D0AH, 041D0AJ, 041D0AK, 041D0AQ, 041D0AR, 041D0J0, 041D0J1, 041D0J2, 041D0J3, 041D0J4, 041D0J5, 041D0J6, 041D0J7, 041D0J8, 041D0J9, 041D0JB, 041D0JC, 041D0JD, 041D0JF, 041D0JG, 041D0JH, 041D0JJ, 041D0JK, 041D0JQ, 041D0JR, 041D0K0, 041D0K1, 041D0K2, 041D0K3, 041D0K4, 041D0K5, 041D0K6, 041D0K7, 041D0K8, 041D0K9, 041D0KB, 041D0KC, 041D0KD, 041D0KF, 041D0KG, 041D0KH, 041D0KJ, 041D0KK, 041D0KQ, 041D0KR, 041D0Z0, 041D0Z1, 041D0Z2, 041D0Z3, 041D0Z4, 041D0Z5, 041D0Z6, 041D0Z7, 041D0Z8, 041D0Z9, 041D0ZB, 041D0ZC, 041D0ZD, 041D0ZF, 041D0ZG, 041D0ZH, 041D0ZJ, 041D0ZK, 041D0ZQ, 041D0ZR, 041D490, 041D491, 041D492, 041D493, 041D494, 041D495, 041D496, 041D497, 041D498, 041D499, 041D49B, 041D49C, 041D49D, 041D49F, 041D49G, 041D49H, 041D49J, 041D49K, 041D49Q, 041D49R, 041D4A0, 041D4A1, 041D4A2, 041D4A3, 041D4A4, 041D4A5, 041D4A6, 041D4A7, 041D4A8, 041D4A9, 041D4AB, 041D4AC, 041D4AD, 041D4AF, 041D4AG, 041D4AH, 041D4AJ, 041D4AK, 041D4AQ, 041D4AR, 041D4J0, 041D4J1, 041D4J2, 041D4J3, 041D4J4, 041D4J5, 041D4J6, 041D4J7, 041D4J8, 041D4J9, 041D4JB, 041D4JC, 041D4JD, 041D4JF, 041D4JG, 041D4JH, 041D4JJ, 041D4JK, 041D4JQ,

© 2022 Stewart IJ et al. *JAMA Neurology.*

		041D4JR, 041D4K0, 041D4K1, 041D4K2, 041D4K3, 041D4K4, 041D4K5, 041D4K6, 041D4K7, 041D4K8, 041D4K9, 041D4KB, 041D4KC, 041D4KD, 041D4KF, 041D4KG, 041D4KH, 041D4KJ, 041D4KK, 041D4KQ, 041D4KR, 041D4Z0, 041D4Z1, 041D4Z2, 041D4Z3, 041D4Z4, 041D4Z5, 041D4Z6, 041D4Z7, 041D4Z8, 041D4Z9, 041D4ZB, 041D4ZC, 041D4ZD, 041D4ZF, 041D4ZG, 041D4ZH, 041D4ZJ, 041D4ZK, 041D4ZQ, 041D4ZR,041E099, 041E09B, 041E09C, 041E09D, 041E09F, 041E09G, 041E09H, 041E09J, 041E09K, 041E09P, 041E09Q, 041E0A9, 041E0AB, 041E0AC, 041E0AD, 041E0AF, 041E0AG, 041E0AH, 041E0AJ, 041E0AK, 041E0AP, 041E0AQ, 041E0J9, 041E0JB, 041E0JC, 041E0JD, 041E0JF, 041E0JG, 041E0JH, 041E0JJ, 041E0JK, 041E0JP, 041E0JQ, 041E0K9, 041E0KB, 041E0KC, 041E0KD, 041E0KF, 041E0KG, 041E0KH, 041E0KJ, 041E0KK, 041E0KP, 041E0KQ, 041E0Z9, 041E0ZB, 041E0ZC, 041E0ZD, 041E0ZF, 041E0ZG, 041E0ZH, 041E0ZJ, 041E0ZK, 041E0ZP, 041E0ZQ, 041E499, 041E49B, 041E49C, 041E49D, 041E49F, 041E49G, 041E49H, 041E49J, 041E49K, 041E49P, 041E49Q, 041E4A9, 041E4AB, 041E4AC, 041E4AD, 041E4AF, 041E4AG, 041E4AH, 041E4AJ, 041E4AK, 041E4AP, 041E4AQ, 041E4J9, 041E4JB, 041E4JC, 041E4JD, 041E4JF, 041E4JG, 041E4JH, 041E4JJ, 041E4JK, 041E4JP, 041E4JQ, 041E4K9, 041E4KB, 041E4KC, 041E4KD, 041E4KF, 041E4KG, 041E4KH, 041E4KJ, 041E4KK, 041E4KP, 041E4KQ, 041E4Z9, 041E4ZB, 041E4ZC, 041E4ZD, 041E4ZF, 041E4ZG, 041E4ZH, 041E4ZJ, 041E4ZK, 041E4ZP, 041E4ZQ, 041F099, 041F09B, 041F09C, 041F09D, 041F09F, 041F09G, 041F09H, 041F09J, 041F09K, 041F09P, 041F09Q, 041F0A9, 041F0AB, 041F0AC, 041F0AD, 041F0AF, 041F0AG,

© 2022 Stewart IJ et al. *JAMA Neurology.*

		041F0AH, 041F0AJ, 041F0AK, 041F0AP, 041F0AQ, 041F0J9, 041F0JB, 041F0JC, 041F0JD, 041F0JF, 041F0JG, 041F0JH, 041F0JJ, 041F0JK, 041F0JP, 041F0JQ, 041F0K9, 041F0KB, 041F0KC, 041F0KD, 041F0KF, 041F0KG, 041F0KH, 041F0KJ, 041F0KK, 041F0KP, 041F0KQ, 041F0Z9, 041F0ZB, 041F0ZC, 041F0ZD, 041F0ZF, 041F0ZG, 041F0ZH, 041F0ZJ, 041F0ZK, 041F0ZP, 041F0ZQ, 041F499, 041F49B, 041F49C, 041F49D, 041F49F, 041F49G, 041F49H, 041F49J, 041F49K, 041F49P, 041F49Q, 041F4A9, 041F4AB, 041F4AC, 041F4AD, 041F4AF, 041F4AG, 041F4AH, 041F4AJ, 041F4AK, 041F4AP, 041F4AQ, 041F4J9, 041F4JB, 041F4JC, 041F4JD, 041F4JF, 041F4JG, 041F4JH, 041F4JJ, 041F4JK, 041F4JP, 041F4JQ, 041F4K9, 041F4KB, 041F4KC, 041F4KD, 041F4KF, 041F4KG, 041F4KH, 041F4KJ, 041F4KK, 041F4KP, 041F4KQ, 041F4Z9, 041F4ZB, 041F4ZC, 041F4ZD, 041F4ZF, 041F4ZG, 041F4ZH, 041F4ZJ, 041F4ZK, 041F4ZP, 041F4ZQ, 041H099, 041H09B, 041H09C, 041H09D, 041H09F, 041H09G, 041H09H, 041H09J, 041H09K, 041H09P, 041H09Q, 041H0A9, 041H0AB, 041H0AC, 041H0AD, 041H0AF, 041H0AG, 041H0AH, 041H0AJ, 041H0AK, 041H0AP, 041H0AQ, 041H0J9, 041H0JB, 041H0JC, 041H0JD, 041H0JF, 041H0JG, 041H0JH, 041H0JJ, 041H0JK, 041H0JP, 041H0JQ, 041H0K9, 041H0KB, 041H0KC, 041H0KD, 041H0KF, 041H0KG, 041H0KH, 041H0KJ, 041H0KK, 041H0KP, 041H0KQ, 041H0Z9, 041H0ZB, 041H0ZC, 041H0ZD, 041H0ZF, 041H0ZG, 041H0ZH, 041H0ZJ, 041H0ZK, 041H0ZP, 041H0ZQ, 041H499, 041H49B, 041H49C, 041H49D, 041H49F, 041H49G, 041H49H, 041H49J, 041H49K, 041H49P, 041H49Q, 041H4A9, 041H4AB, 041H4AC, 041H4AD, 041H4AF, 041H4AG, 041H4AH, 041H4AJ, 041H4AK,

© 2022 Stewart IJ et al. *JAMA Neurology.*

		041H4AP, 041H4AQ, 041H4J9, 041H4JB, 041H4JC, 041H4JD, 041H4JF, 041H4JG, 041H4JH, 041H4JJ, 041H4JK, 041H4JP, 041H4JQ, 041H4K9, 041H4KB, 041H4KC, 041H4KD, 041H4KF, 041H4KG, 041H4KH, 041H4KJ, 041H4KK, 041H4KP, 041H4KQ, 041H4Z9, 041H4ZB, 041H4ZC, 041H4ZD, 041H4ZF, 041H4ZG, 041H4ZH, 041H4ZJ, 041H4ZK, 041H4ZP, 041H4ZQ, 041J099, 041J09B, 041J09C, 041J09D, 041J09F, 041J09G, 041J09H, 041J09J, 041J09K, 041J09P, 041J09Q, 041J0A9, 041J0AB, 041J0AC, 041J0AD, 041J0AF, 041J0AG, 041J0AH, 041J0AJ, 041J0AK, 041J0AP, 041J0AQ, 041J0J9, 041J0JB, 041J0JC, 041J0JD, 041J0JF, 041J0JG, 041J0JH, 041J0JJ, 041J0JK, 041J0JP, 041J0JQ, 041J0K9, 041J0KB, 041J0KC, 041J0KD, 041J0KF, 041J0KG, 041J0KH, 041J0KJ, 041J0KK, 041J0KP, 041J0KQ, 041J0Z9, 041J0ZB, 041J0ZC, 041J0ZD, 041J0ZF, 041J0ZG, 041J0ZH, 041J0ZJ, 041J0ZK, 041J0ZP, 041J0ZQ, 041J499, 041J49B, 041J49C, 041J49D, 041J49F, 041J49G, 041J49H, 041J49J, 041J49K, 041J49P, 041J49Q, 041J4A9, 041J4AB, 041J4AC, 041J4AD, 041J4AF, 041J4AG, 041J4AH, 041J4AJ, 041J4AK, 041J4AP, 041J4AQ, 041J4J9, 041J4JB, 041J4JC, 041J4JD, 041J4JF, 041J4JG, 041J4JH, 041J4JJ, 041J4JK, 041J4JP, 041J4JQ, 041J4K9, 041J4KB, 041J4KC, 041J4KD, 041J4KF, 041J4KG, 041J4KH, 041J4KJ, 041J4KK, 041J4KP, 041J4KQ, 041J4Z9, 041J4ZB, 041J4ZC, 041J4ZD, 041J4ZF, 041J4ZG, 041J4ZH, 041J4ZJ, 041J4ZK, 041J4ZP, 041J4ZQ, 049Y0ZZ, 049Y3ZZ, 049Y4ZZ, 04C00ZZ, 04C03ZZ, 04C04ZZ,04C10ZZ, 04C13ZZ, 04C14ZZ, 04C20ZZ, 04C23ZZ, 04C24ZZ, 04C30ZZ, 04C33ZZ, 04C34ZZ, 04C40ZZ, 04C43ZZ, 04C44ZZ, 04C50ZZ, 04C53ZZ, 04C54ZZ, 04C60ZZ, 04C63ZZ,

© 2022 Stewart IJ et al. *JAMA Neurology.*

		04C64ZZ, 04C70ZZ, 04C73ZZ, 04C74ZZ, 04C80ZZ, 04C83ZZ, 04C84ZZ, 04C90ZZ, 04C93ZZ, 04C94ZZ, 04CA0ZZ, 04CA3ZZ, 04CA4ZZ, 04CB0ZZ, 04CB3ZZ, 04CB4ZZ, 04CC0ZZ, 04CC3ZZ, 04CC4ZZ, 04CD0ZZ, 04CD3ZZ, 04CD4ZZ, 04CE0ZZ, 04CE3ZZ, 04CE4ZZ, 04CF0ZZ, 04CF3ZZ, 04CF4ZZ, 04CH0ZZ, 04CH3ZZ, 04CH4ZZ, 04CJ0ZZ, 04CJ3ZZ, 04CJ4ZZ, 04CK0ZZ, 04CK3ZZ, 04CK4ZZ, 04CL0ZZ, 04CL3ZZ, 04CL4ZZ, 04CM0ZZ, 04CM3ZZ, 04CM4ZZ, 04CN0ZZ, 04CN3ZZ, 04CN4ZZ, 04CP0ZZ, 04CP3ZZ, 04CP4ZZ, 04CQ0ZZ, 04CQ3ZZ, 04CQ4ZZ, 04CR0ZZ, 04CR3ZZ, 04CR4ZZ, 04CS0ZZ, 04CS3ZZ, 04CS4ZZ, 04CT0ZZ, 04CT3ZZ, 04CT4ZZ, 04CU0ZZ, 04CU3ZZ, 04CU4ZZ, 04CV0ZZ, 04CV3ZZ, 04CV4ZZ, 04CW0ZZ, 04CW3ZZ, 04CW4ZZ, 04CY0ZZ, 04CY3ZZ, 04CY4ZZ, 04HY02Z, 04HY32Z, 04HY42Z, 051007Y, 051009Y, 05100AY, 05100JY, 05100KY, 05100ZY, 051047Y, 051049Y, 05104AY, 05104JY, 05104KY, 05104ZY, 051107Y, 051109Y, 05110AY, 05110JY, 05110KY, 05110ZY, 051147Y, 051149Y, 05114AY, 05114JY, 05114KY, 05114ZY, 051307Y, 051309Y, 05130AY, 05130JY, 05130KY, 05130ZY, 051347Y, 051349Y, 05134AY, 05134JY, 05134KY, 05134ZY, 051407Y, 051409Y, 05140AY, 05140JY, 05140KY, 05140ZY, 051447Y, 051449Y, 05144AY, 05144JY, 05144KY, 05144ZY, 051507Y, 051509Y, 05150AY, 05150JY, 05150KY, 05150ZY, 051547Y, 051549Y, 05154AY, 05154JY, 05154KY, 05154ZY, 051607Y, 051609Y, 05160AY, 05160JY, 05160KY, 05160ZY, 051647Y, 051649Y, 05164AY, 05164JY, 05164KY, 05164ZY, 059Y00Z, 059Y0ZZ, 059Y30Z, 059Y3ZZ, 05C00ZZ, 05C03ZZ, 05C04ZZ, 05C10ZZ, 05C13ZZ, 05C14ZZ, 05C30ZZ, 05C33ZZ, 05C34ZZ, 05C40ZZ, 05C43ZZ, 05C44ZZ, 05C50ZZ, 05C53ZZ, 05C54ZZ, 05C60ZZ,

© 2022 Stewart IJ et al. *JAMA Neurology.*

		05C63ZZ, 05C64ZZ, 05C70ZZ, 05C73ZZ, 05C74ZZ, 05C80ZZ, 05C83ZZ, 05C84ZZ, 05C90ZZ, 05C93ZZ, 05C94ZZ, 05CA0ZZ, 05CA3ZZ, 05CA4ZZ, 05CB0ZZ, 05CB3ZZ, 05CB4ZZ, 05CC0ZZ, 05CC3ZZ, 05CC4ZZ, 05CD0ZZ, 05CD3ZZ, 05CD4ZZ, 05CF0ZZ, 05CF3ZZ, 05CF4ZZ, 05CG0ZZ, 05CG3ZZ, 05CG4ZZ, 05CH0ZZ, 05CH3ZZ, 05CH4ZZ, 05CL0ZZ, 05CL4ZZ, 05CM0ZZ, 05CM3ZZ, 05CM4ZZ, 05CN0ZZ, 05CN3ZZ, 05CN4ZZ, 05CP0ZZ, 05CP3ZZ, 05CP4ZZ, 05CQ0ZZ, 05CQ3ZZ, 05CQ4ZZ, 05CR0ZZ, 05CR3ZZ, 05CR4ZZ, 05CS0ZZ, 05CS3ZZ, 05CS4ZZ, 05CT0ZZ, 05CT3ZZ, 05CT4ZZ, 05CV0ZZ, 05CV3ZZ, 05CV4ZZ, 05CY0ZZ, 05CY3ZZ, 05CY4ZZ, 05HY02Z, 05HY32Z, 05HY42Z, 069300Z, 06930ZZ, 069330Z, 06933ZZ, 069340Z, 06934ZZ, 069Y00Z, 069Y0ZZ, 069Y30Z, 069Y3ZZ, 06C00ZZ, 06C03ZZ, 06C04ZZ, 06C10ZZ, 06C13ZZ, 06C14ZZ, 06C20ZZ, 06C23ZZ, 06C24ZZ, 06C30ZZ, 06C33ZZ, 06C34ZZ, 06C40ZZ, 06C43ZZ, 06C44ZZ, 06C50ZZ, 06C53ZZ, 06C54ZZ, 06C60ZZ, 06C63ZZ, 06C64ZZ, 06C70ZZ, 06C73ZZ, 06C74ZZ, 06C80ZZ, 06C83ZZ, 06C84ZZ, 06C90ZZ, 06C93ZZ, 06C94ZZ, 06CB0ZZ, 06CB3ZZ, 06CB4ZZ, 06CC0ZZ, 06CC3ZZ, 06CC4ZZ, 06CD0ZZ, 06CD3ZZ, 06CD4ZZ, 06CF0ZZ, 06CF3ZZ, 06CF4ZZ, 06CG0ZZ, 06CG3ZZ, 06CG4ZZ, 06CH0ZZ, 06CH3ZZ, 06CH4ZZ, 06CJ0ZZ, 06CJ3ZZ, 06CJ4ZZ, 06CM0ZZ, 06CM3ZZ, 06CM4ZZ, 06CN0ZZ, 06CN3ZZ, 06CN4ZZ, 06CP0ZZ, 06CP3ZZ, 06CP4ZZ, 06CQ0ZZ, 06CQ3ZZ, 06CQ4ZZ, 06CR0ZZ, 06CR3ZZ, 06CR4ZZ, 06CS0ZZ, 06CS3ZZ, 06CS4ZZ, 06CT0ZZ, 06CT3ZZ, 06CT4ZZ, 06CV0ZZ, 06CV3ZZ, 06CV4ZZ, 06CY0ZZ, 06CY3ZZ, 06CY4ZZ, 06HY02Z, 06HY32Z, 06HY42Z, 0210098, 0210099, 021009C, 021009F, 021009W, 02100A3, 02100A8, 02100A9, 02100AC, 02100AF, 02100AW, 02100J3, 02100J8,

© 2022 Stewart IJ et al. *JAMA Neurology.*

		02100J9, 02100JC, 02100JF, 02100JW, 02100K3, 02100K8, 02100K9, 02100KC, 02100KF, 02100KW, 02100Z3, 02100Z8, 02100Z9, 02100ZC, 02100ZF, 0210493, 0210498, 0210499, 021049C, 021049F, 021049W, 02104A3, 02104A8, 02104A9, 02104AC, 02104AF, 02104AW, 02104J3, 02104J8, 02104J9, 02104JC, 02104JF, 02104JW, 02104K3, 02104K8, 02104K9, 02104KC, 02104KF, 02104KW, 02104Z3, 02104Z8, 02104Z9, 02104ZC, 02104ZF, 0211098, 0211099, 021109C, 021109W, 02110A8, 02110A9, 02110AC, 02110AW, 02110J8, 02110J9, 02110JC, 02110JW, 02110K8, 02110K9, 02110KC, 02110KW, 02110Z8, 02110Z9, 02110ZC, 0211498, 0211499, 021149C, 021149W, 02114A8, 02114A9, 02114AC, 02114AW, 02114J8, 02114J9, 02114JC, 02114JW, 02114K8, 02114K9, 02114KC, 02114KW, 02114Z8, 02114Z9, 02114ZC, 021209C, 021209W, 02120AC, 02120AW, 02120JC, 02120JW, 02120KC, 02120KW, 02120ZC, 021249C, 021249W, 02124AC, 02124AW, 02124JC, 02124JW, 02124KC, 02124KW, 02124ZC, 021309C, 021309W, 02130AC, 02130AW, 02130JC, 02130JW, 02130KC, 02130KW, 02130ZC, 021349C, 021349W, 02134AC, 02134AW, 02134JC, 02134JW, 02134KC, 02134KW, 02134ZC, 021K0Z8, 021K0Z9, 021K0ZC, 021K0ZW, 021K4Z8, 021K4Z9, 021K4ZC, 021K4ZW, 021L0Z8, 021L0Z9, 021L0ZC, 021L4Z8, 021L4Z9, 021L4ZC, 02700ZZ, 02710ZZ, 02720ZZ, 02730ZZ, 02C00ZZ, 02C03ZZ, 02C04ZZ, 02C10ZZ, 02C13ZZ, 02C14ZZ, 02C20ZZ, 02C23ZZ, 02C24ZZ, 02C30ZZ, 02C33ZZ, 02C34ZZ, 3E07017, 3E070PZ, 3E07317, 3E073PZ<br><br>Peripheral arterial procedures<br>021W09B, 021W09D, 021W0AB, 021W0AD, 021W0JB, 021W0JD,

© 2022 Stewart IJ et al. *JAMA Neurology.*

		021W0KB, 021W0KD, 021W0ZB, 021W0ZD, 021W49B, 021W49D, 021W4AB, 021W4AD, 021W4JB, 021W4JD, 021W4KB, 021W4KD, 021W4ZB, 021W4ZD, 02CP0ZZ, 02CP3ZZ, 02CP4ZZ, 02CQ0ZZ, 02CQ3ZZ, 02CQ4ZZ, 02CR0ZZ, 02CR3ZZ, 02CR4ZZ, 02CS0ZZ, 02CS3ZZ, 02CS4ZZ, 02CT0ZZ, 02CT3ZZ, 02CT4ZZ, 02CV0ZZ, 02CV3ZZ, 02CV4ZZ, 02CW0ZZ, 02CW3ZZ, 02CW4ZZ, 02HP0DZ, 02HP3DZ, 02HP4DZ, 02HQ0DZ, 02HQ3DZ, 02HQ4DZ, 02HR0DZ, 02HR3DZ, 02HR4DZ, 02HS02Z, 02HS0DZ, 02HS32Z, 02HS3DZ, 02HS42Z, 02HS4DZ, 02HT02Z, 02HT0DZ, 02HT32Z, 02HT3DZ, 02HT42Z, 02HT4DZ, 02HW02Z, 02HW0DZ, 02HW32Z, 02HW3DZ, 02HW42Z, 02HW4DZ, 031H09J, 031H0AJ, 031H0JJ, 031H0KJ, 031H0ZJ, 031J09K, 031J0AK, 031J0JK, 031J0KK, 031J0ZK, 031K09J, 031K0AJ, 031K0JJ, 031K0KJ, 031K0ZJ, 031L09K, 031L0AK, 031L0JK, 031L0KK, 031L0ZK, 031M09J, 031M0AJ, 031M0JJ, 031M0KJ, 031M0ZJ, 031N09K, 031N0AK, 031N0JK, 031N0KK, 031N0ZK, 031S09G, 031S0AG, 031S0JG, 031S0KG, 031S0ZG, 031T09G, 031T0AG, 031T0JG, 031T0KG, 031T0ZG, 039Y0ZZ, 039Y3ZZ, 039Y4ZZ, 03C00ZZ, 03C03ZZ, 03C04ZZ, 03C10ZZ, 03C13ZZ, 03C14ZZ, 03C20ZZ, 03C23ZZ, 03C24ZZ, 03C30ZZ, 03C33ZZ, 03C34ZZ, 03C40ZZ, 03C43ZZ, 03C44ZZ, 03C50ZZ, 03C53ZZ, 03C54ZZ, 03C60ZZ, 03C63ZZ, 03C64ZZ, 03C70ZZ, 03C73ZZ, 03C74ZZ, 03C80ZZ, 03C83ZZ, 03C84ZZ, 03C90ZZ, 03C93ZZ, 03C94ZZ, 03CA0ZZ, 03CA3ZZ, 03CA4ZZ, 03CB0ZZ, 03CB3ZZ, 03CB4ZZ, 03CC0ZZ, 03CC3ZZ, 03CC4ZZ, 03CD0ZZ, 03CD3ZZ, 03CD4ZZ, 03CF0ZZ, 03CF3ZZ, 03CF4ZZ, 03CG0ZZ, 03CG4ZZ, 03CH0ZZ, 03CH4ZZ, 03CJ0ZZ, 03CJ4ZZ, 03CK0ZZ, 03CK4ZZ, 03CL0ZZ, 03CL4ZZ, 03CM0ZZ, 03CM4ZZ, 03CN0ZZ,

© 2022 Stewart IJ et al. *JAMA Neurology.*

		03CN4ZZ, 03CP0ZZ, 03CP4ZZ, 03CQ0ZZ, 03CQ4ZZ, 03CR0ZZ, 03CR3ZZ, 03CR4ZZ, 03CS0ZZ, 03CS3ZZ, 03CS4ZZ, 03CT0ZZ, 03CT3ZZ, 03CT4ZZ, 03CU0ZZ, 03CU3ZZ, 03CU4ZZ, 03CV0ZZ, 03CV3ZZ, 03CV4ZZ, 03CY0ZZ, 03CY3ZZ, 03CY4ZZ, 03HY02Z, 03HY32Z, 03HY42Z, 0410090, 0410091, 0410092, 0410093, 0410094, 0410095, 0410096, 0410097, 0410098, 0410099, 041009B, 041009C, 041009D, 041009F, 041009G, 041009H, 041009J, 041009K, 041009Q, 041009R, 04100A0, 04100A1, 04100A2, 04100A3, 04100A4, 04100A5, 04100A6, 04100A7, 04100A8, 04100A9, 04100AB, 04100AC, 04100AD, 04100AF, 04100AG, 04100AH, 04100AJ, 04100AK, 04100AQ, 04100AR, 04100J0, 04100J1, 04100J2, 04100J3, 04100J4, 04100J5, 04100J6, 04100J7, 04100J8, 04100J9, 04100JB, 04100JC, 04100JD, 04100JF, 04100JG, 04100JH, 04100JJ, 04100JK, 04100JQ, 04100JR, 04100K0, 04100K1, 04100K2, 04100K3, 04100K4, 04100K5, 04100K6, 04100K7, 04100K8, 04100K9, 04100KB, 04100KC, 04100KD, 04100KF, 04100KG, 04100KH, 04100KJ, 04100KK, 04100KQ, 04100KR, 04100Z0, 04100Z1, 04100Z2, 04100Z3, 04100Z4, 04100Z5, 04100Z6, 04100Z7, 04100Z8, 04100Z9, 04100ZB, 04100ZC, 04100ZD, 04100ZF, 04100ZG, 04100ZH, 04100ZJ, 04100ZK, 04100ZQ, 04100ZR, 0410490, 0410491, 0410492, 0410493, 0410494, 0410495, 0410496, 0410497, 0410498, 0410499, 041049B, 041049C, 041049D, 041049F, 041049G, 041049H, 041049J, 041049K, 041049Q, 041049R, 04104A0, 04104A1, 04104A2, 04104A3, 04104A4, 04104A5, 04104A6, 04104A7, 04104A8, 04104A9, 04104AB, 04104AC, 04104AD, 04104AF, 04104AG, 04104AH, 04104AJ, 04104AK, 04104AQ, 04104AR, 04104J0, 04104J1,

© 2022 Stewart IJ et al. *JAMA Neurology.*

		04104J2, 04104J3, 04104J4, 04104J5, 04104J6, 04104J7, 04104J8, 04104J9, 04104JB, 04104JC, 04104JD, 04104JF, 04104JG, 04104JH, 04104JJ, 04104JK, 04104JQ, 04104JR, 04104K0, 04104K1, 04104K2, 04104K3, 04104K4, 04104K5, 04104K6, 04104K7, 04104K8, 04104K9, 04104KB, 04104KC, 04104KD, 04104KF, 04104KG, 04104KH, 04104KJ, 04104KK, 04104KQ, 04104KR, 04104Z0, 04104Z1, 04104Z2, 04104Z3, 04104Z4, 04104Z5, 04104Z6, 04104Z7, 04104Z8, 04104Z9, 04104ZB, 04104ZC, 04104ZD, 04104ZF, 04104ZG, 04104ZH, 04104ZJ, 04104ZK, 04104ZQ, 04104ZR, 0414093, 0414094, 0414095, 04140A3, 04140A4, 04140A5, 04140J3, 04140J4, 04140J5, 04140K3, 04140K4, 04140K5, 04140Z3, 04140Z4, 04140Z5, 0414493, 0414494, 0414495, 04144A3,04144A4, 04144A5, 04144J3, 04144J4, 04144J5, 04144K3, 04144K4, 04144K5, 04144Z3,04144Z4, 04144Z5, 041C090, 041C091, 041C092, 041C093, 041C094, 041C095, 041C096, 041C097, 041C098, 041C099, 041C09B, 041C09C, 041C09D, 041C09F, 041C09G, 041C09H, 041C09J, 041C09K, 041C09Q, 041C09R, 041C0A0, 041C0A1, 041C0A2, 041C0A3, 041C0A4, 041C0A5, 041C0A6, 041C0A7, 041C0A8, 041C0A9, 041C0AB, 041C0AC, 041C0AD, 041C0AF, 041C0AG, 041C0AH, 041C0AJ, 041C0AK, 041C0AQ, 041C0AR, 041C0J0, 041C0J1, 041C0J2, 041C0J3, 041C0J4, 041C0J5, 041C0J6, 041C0J7, 041C0J8, 041C0J9, 041C0JB, 041C0JC, 041C0JD, 041C0JF, 041C0JG, 041C0JH, 041C0JJ, 041C0JK, 041C0JQ, 041C0JR, 041C0K0, 041C0K1, 041C0K2, 041C0K3, 041C0K4, 041C0K5, 041C0K6, 041C0K7, 041C0K8, 041C0K9, 041C0KB, 041C0KC, 041C0KD, 041C0KF, 041C0KG, 041C0KH, 041C0KJ, 041C0KK, 041C0KQ, 041C0KR,

© 2022 Stewart IJ et al. *JAMA Neurology.*

		041C0Z0, 041C0Z1, 041C0Z2, 041C0Z3, 041C0Z4, 041C0Z5, 041C0Z6, 041C0Z7, 041C0Z8, 041C0Z9, 041C0ZB, 041C0ZC, 041C0ZD, 041C0ZF, 041C0ZG, 041C0ZH, 041C0ZJ, 041C0ZK, 041C0ZQ, 041C0ZR, 041C490, 041C491, 041C492, 041C493, 041C494, 041C495, 041C496, 041C497, 041C498, 041C499, 041C49B, 041C49C, 041C49D, 041C49F, 041C49G, 041C49H, 041C49J, 041C49K, 041C49Q, 041C49R, 041C4A0, 041C4A1, 041C4A2, 041C4A3, 041C4A4, 041C4A5, 041C4A6, 041C4A7, 041C4A8, 041C4A9, 041C4AB, 041C4AC, 041C4AD, 041C4AF, 041C4AG, 041C4AH, 041C4AJ, 041C4AK, 041C4AQ, 041C4AR, 041C4J0, 041C4J1, 041C4J2, 041C4J3, 041C4J4, 041C4J5, 041C4J6, 041C4J7, 041C4J8, 041C4J9, 041C4JB, 041C4JC, 041C4JD, 041C4JF, 041C4JG, 041C4JH, 041C4JJ, 041C4JK, 041C4JQ, 041C4JR, 041C4K0, 041C4K1, 041C4K2, 041C4K3, 041C4K4, 041C4K5, 041C4K6, 041C4K7, 041C4K8, 041C4K9, 041C4KB, 041C4KC, 041C4KD, 041C4KF, 041C4KG, 041C4KH, 041C4KJ, 041C4KK, 041C4KQ, 041C4KR, 041C4Z0, 041C4Z1, 041C4Z2, 041C4Z3, 041C4Z4, 041C4Z5, 041C4Z6, 041C4Z7, 041C4Z8, 041C4Z9, 041C4ZB, 041C4ZC, 041C4ZD, 041C4ZF, 041C4ZG, 041C4ZH, 041C4ZJ, 041C4ZK, 041C4ZQ, 041C4ZR, 041D090, 041D091, 041D092, 041D093, 041D094, 041D095, 041D096, 041D097, 041D098, 041D099, 041D09B, 041D09C, 041D09D, 041D09F, 041D09G, 041D09H, 041D09J, 041D09K, 041D09Q, 041D09R, 041D0A0, 041D0A1, 041D0A2, 041D0A3, 041D0A4, 041D0A5, 041D0A6, 041D0A7, 041D0A8, 041D0A9, 041D0AB, 041D0AC, 041D0AD, 041D0AF, 041D0AG, 041D0AH, 041D0AJ, 041D0AK, 041D0AQ, 041D0AR, 041D0J0, 041D0J1, 041D0J2, 041D0J3, 041D0J4, 041D0J5, 041D0J6, 041D0J7,

© 2022 Stewart IJ et al. *JAMA Neurology.*

		041D0J8, 041D0J9, 041D0JB, 041D0JC, 041D0JD, 041D0JF, 041D0JG, 041D0JH, 041D0JJ, 041D0JK, 041D0JQ, 041D0JR, 041D0K0, 041D0K1, 041D0K2, 041D0K3, 041D0K4, 041D0K5, 041D0K6, 041D0K7, 041D0K8, 041D0K9, 041D0KB, 041D0KC, 041D0KD, 041D0KF, 041D0KG, 041D0KH, 041D0KJ, 041D0KK, 041D0KQ, 041D0KR, 041D0Z0, 041D0Z1, 041D0Z2, 041D0Z3, 041D0Z4, 041D0Z5, 041D0Z6, 041D0Z7, 041D0Z8, 041D0Z9, 041D0ZB, 041D0ZC, 041D0ZD, 041D0ZF, 041D0ZG, 041D0ZH, 041D0ZJ, 041D0ZK, 041D0ZQ, 041D0ZR, 041D490, 041D491, 041D492, 041D493, 041D494, 041D495, 041D496, 041D497, 041D498, 041D499, 041D49B, 041D49C, 041D49D, 041D49F, 041D49G, 041D49H, 041D49J, 041D49K, 041D49Q, 041D49R, 041D4A0, 041D4A1, 041D4A2, 041D4A3, 041D4A4, 041D4A5, 041D4A6, 041D4A7, 041D4A8, 041D4A9, 041D4AB, 041D4AC, 041D4AD, 041D4AF, 041D4AG, 041D4AH, 041D4AJ, 041D4AK, 041D4AQ, 041D4AR, 041D4J0, 041D4J1, 041D4J2, 041D4J3, 041D4J4, 041D4J5, 041D4J6, 041D4J7, 041D4J8, 041D4J9, 041D4JB, 041D4JC, 041D4JD, 041D4JF, 041D4JG, 041D4JH, 041D4JJ, 041D4JK, 041D4JQ, 041D4JR, 041D4K0, 041D4K1, 041D4K2, 041D4K3, 041D4K4, 041D4K5, 041D4K6, 041D4K7, 041D4K8, 041D4K9, 041D4KB, 041D4KC, 041D4KD, 041D4KF, 041D4KG, 041D4KH, 041D4KJ, 041D4KK, 041D4KQ, 041D4KR, 041D4Z0, 041D4Z1, 041D4Z2, 041D4Z3, 041D4Z4, 041D4Z5, 041D4Z6, 041D4Z7, 041D4Z8, 041D4Z9, 041D4ZB, 041D4ZC, 041D4ZD, 041D4ZF, 041D4ZG, 041D4ZH, 041D4ZJ, 041D4ZK, 041D4ZQ, 041D4ZR, 041E099, 041E09B, 041E09C, 041E09D, 041E09F, 041E09G, 041E09H, 041E09J, 041E09K, 041E09P, 041E09Q, 041E0A9, 041E0AB, 041E0AC, 041E0AD, 041E0AF,

© 2022 Stewart IJ et al. *JAMA Neurology.*

		041E0AG, 041E0AH, 041E0AJ, 041E0AK, 041E0AP, 041E0AQ, 041E0J9, 041E0JB, 041E0JC, 041E0JD, 041E0JF, 041E0JG, 041E0JH, 041E0JJ, 041E0JK, 041E0JP, 041E0JQ, 041E0K9, 041E0KB, 041E0KC, 041E0KD, 041E0KF, 041E0KG, 041E0KH, 041E0KJ, 041E0KK, 041E0KP, 041E0KQ, 041E0Z9, 041E0ZB, 041E0ZC, 041E0ZD, 041E0ZF, 041E0ZG, 041E0ZH, 041E0ZJ, 041E0ZK, 041E0ZP, 041E0ZQ, 041E499, 041E49B, 041E49C, 041E49D, 041E49F, 041E49G, 041E49H, 041E49J, 041E49K, 041E49P, 041E49Q, 041E4A9, 041E4AB, 041E4AC, 041E4AD, 041E4AF, 041E4AG, 041E4AH, 041E4AJ, 041E4AK, 041E4AP, 041E4AQ, 041E4J9, 041E4JB, 041E4JC, 041E4JD, 041E4JF, 041E4JG, 041E4JH, 041E4JJ, 041E4JK, 041E4JP, 041E4JQ, 041E4K9, 041E4KB, 041E4KC, 041E4KD, 041E4KF, 041E4KG, 041E4KH, 041E4KJ, 041E4KK, 041E4KP, 041E4KQ, 041E4Z9, 041E4ZB, 041E4ZC, 041E4ZD, 041E4ZF, 041E4ZG, 041E4ZH, 041E4ZJ, 041E4ZK, 041E4ZP, 041E4ZQ, 041F099, 041F09B, 041F09C, 041F09D, 041F09F, 041F09G, 041F09H, 041F09J, 041F09K, 041F09P, 041F09Q, 041F0A9, 041F0AB, 041F0AC, 041F0AD, 041F0AF, 041F0AG, 041F0AH, 041F0AJ, 041F0AK, 041F0AP, 041F0AQ, 041F0J9, 041F0JB, 041F0JC, 041F0JD, 041F0JF, 041F0JG, 041F0JH, 041F0JJ, 041F0JK, 041F0JP, 041F0JQ, 041F0K9, 041F0KB, 041F0KC, 041F0KD, 041F0KF, 041F0KG, 041F0KH, 041F0KJ, 041F0KK, 041F0KP, 041F0KQ, 041F0Z9, 041F0ZB, 041F0ZC, 041F0ZD, 041F0ZF, 041F0ZG, 041F0ZH, 041F0ZJ, 041F0ZK, 041F0ZP, 041F0ZQ, 041F499, 041F49B, 041F49C, 041F49D, 041F49F, 041F49G, 041F49H, 041F49J, 041F49K, 041F49P, 041F49Q, 041F4A9, 041F4AB, 041F4AC, 041F4AD, 041F4AF, 041F4AG, 041F4AH, 041F4AJ,

© 2022 Stewart IJ et al. *JAMA Neurology.*

		041F4AK, 041F4AP, 041F4AQ, 041F4J9, 041F4JB, 041F4JC, 041F4JD, 041F4JF, 041F4JG, 041F4JH, 041F4JJ, 041F4JK, 041F4JP, 041F4JQ, 041F4K9, 041F4KB, 041F4KC, 041F4KD, 041F4KF, 041F4KG, 041F4KH, 041F4KJ, 041F4KK, 041F4KP, 041F4KQ, 041F4Z9, 041F4ZB, 041F4ZC, 041F4ZD, 041F4ZF, 041F4ZG, 041F4ZH, 041F4ZJ, 041F4ZK, 041F4ZP, 041F4ZQ, 041H099, 041H09B, 041H09C, 041H09D, 041H09F, 041H09G, 041H09H, 041H09J, 041H09K, 041H09P, 041H09Q, 041H0A9, 041H0AB, 041H0AC, 041H0AD, 041H0AF, 041H0AG, 041H0AH, 041H0AJ, 041H0AK, 041H0AP, 041H0AQ, 041H0J9, 041H0JB, 041H0JC, 041H0JD, 041H0JF, 041H0JG, 041H0JH, 041H0JJ, 041H0JK, 041H0JP, 041H0JQ, 041H0K9, 041H0KB, 041H0KC, 041H0KD, 041H0KF, 041H0KG, 041H0KH, 041H0KJ, 041H0KK, 041H0KP, 041H0KQ, 041H0Z9, 041H0ZB, 041H0ZC, 041H0ZD, 041H0ZF, 041H0ZG, 041H0ZH, 041H0ZJ, 041H0ZK, 041H0ZP, 041H0ZQ, 041H499, 041H49B, 041H49C, 041H49D, 041H49F, 041H49G, 041H49H, 041H49J, 041H49K, 041H49P, 041H49Q, 041H4A9, 041H4AB, 041H4AC, 041H4AD, 041H4AF, 041H4AG, 041H4AH, 041H4AJ, 041H4AK, 041H4AP, 041H4AQ, 041H4J9, 041H4JB, 041H4JC, 041H4JD, 041H4JF, 041H4JG, 041H4JH, 041H4JJ, 041H4JK, 041H4JP, 041H4JQ, 041H4K9, 041H4KB, 041H4KC, 041H4KD, 041H4KF, 041H4KG, 041H4KH, 041H4KJ, 041H4KK, 041H4KP, 041H4KQ, 041H4Z9, 041H4ZB, 041H4ZC, 041H4ZD, 041H4ZF, 041H4ZG, 041H4ZH, 041H4ZJ, 041H4ZK, 041H4ZP, 041H4ZQ, 041J099, 041J09B, 041J09C, 041J09D, 041J09F, 041J09G, 041J09H, 041J09J, 041J09K, 041J09P, 041J09Q, 041J0A9, 041J0AB, 041J0AC, 041J0AD, 041J0AF, 041J0AG, 041J0AH, 041J0AJ, 041J0AK, 041J0AP, 041J0AQ,

© 2022 Stewart IJ et al. *JAMA Neurology.*

		041J0J9, 041J0JB, 041J0JC, 041J0JD, 041J0JF, 041J0JG, 041J0JH, 041J0JJ, 041J0JK, 041J0JP, 041J0JQ, 041J0K9, 041J0KB, 041J0KC, 041J0KD, 041J0KF, 041J0KG, 041J0KH, 041J0KJ, 041J0KK, 041J0KP, 041J0KQ, 041J0Z9, 041J0ZB, 041J0ZC, 041J0ZD, 041J0ZF, 041J0ZG, 041J0ZH, 041J0ZJ, 041J0ZK, 041J0ZP, 041J0ZQ, 041J499, 041J49B, 041J49C, 041J49D, 041J49F, 041J49G, 041J49H, 041J49J, 041J49K, 041J49P, 041J49Q, 041J4A9, 041J4AB, 041J4AC, 041J4AD, 041J4AF, 041J4AG, 041J4AH, 041J4AJ, 041J4AK, 041J4AP, 041J4AQ, 041J4J9, 041J4JB, 041J4JC, 041J4JD, 041J4JF, 041J4JG, 041J4JH, 041J4JJ, 041J4JK, 041J4JP, 041J4JQ, 041J4K9, 041J4KB, 041J4KC, 041J4KD, 041J4KF, 041J4KG, 041J4KH, 041J4KJ, 041J4KK, 041J4KP, 041J4KQ, 041J4Z9, 041J4ZB, 041J4ZC, 041J4ZD, 041J4ZF, 041J4ZG, 041J4ZH, 041J4ZJ, 041J4ZK, 041J4ZP, 041J4ZQ, 049Y0ZZ, 049Y3ZZ, 049Y4ZZ, 04C00ZZ, 04C03ZZ, 04C04ZZ, 04C10ZZ, 04C13ZZ, 04C14ZZ, 04C20ZZ, 04C23ZZ, 04C24ZZ, 04C30ZZ, 04C33ZZ, 04C34ZZ, 04C40ZZ, 04C43ZZ, 04C44ZZ, 04C50ZZ, 04C53ZZ, 04C54ZZ, 04C60ZZ, 04C63ZZ, 04C64ZZ, 04C70ZZ, 04C73ZZ, 04C74ZZ, 04C80ZZ, 04C83ZZ, 04C84ZZ, 04C90ZZ, 04C93ZZ, 04C94ZZ, 04CA0ZZ, 04CA3ZZ, 04CA4ZZ, 04CB0ZZ, 04CB3ZZ, 04CB4ZZ, 04CC0ZZ, 04CC3ZZ, 04CC4ZZ, 04CD0ZZ, 04CD3ZZ, 04CD4ZZ, 04CE0ZZ, 04CE3ZZ, 04CE4ZZ, 04CF0ZZ, 04CF3ZZ, 04CF4ZZ, 04CH0ZZ, 04CH3ZZ, 04CH4ZZ, 04CJ0ZZ, 04CJ3ZZ, 04CJ4ZZ, 04CK0ZZ, 04CK3ZZ, 04CK4ZZ, 04CL0ZZ, 04CL3ZZ, 04CL4ZZ, 04CM0ZZ, 04CM3ZZ, 04CM4ZZ, 04CN0ZZ, 04CN3ZZ, 04CN4ZZ, 04CP0ZZ, 04CP3ZZ, 04CP4ZZ, 04CQ0ZZ, 04CQ3ZZ, 04CQ4ZZ, 04CR0ZZ, 04CR3ZZ, 04CR4ZZ, 04CS0ZZ, 04CS3ZZ,

© 2022 Stewart IJ et al. *JAMA Neurology.*

		04CS4ZZ, 04CT0ZZ, 04CT3ZZ,<br>04CT4ZZ, 04CU0ZZ, 04CU3ZZ,<br>04CU4ZZ, 04CV0ZZ, 04CV3ZZ,<br>04CV4ZZ, 04CW0ZZ, 04CW3ZZ,<br>04CW4ZZ, 04CY0ZZ, 04CY3ZZ,<br>04CY4ZZ, 04HY02Z, 04HY32Z,<br>04HY42Z, 051007Y, 051009Y,<br>05100AY, 05100JY, 05100KY,<br>05100ZY, 051047Y, 051049Y,<br>05104AY, 05104JY, 05104KY,<br>05104ZY, 051107Y, 051109Y,<br>05110AY, 05110JY, 05110KY,<br>05110ZY, 051147Y, 051149Y,<br>05114AY, 05114JY, 05114KY,<br>05114ZY, 051307Y, 051309Y,<br>05130AY, 05130JY, 05130KY,<br>05130ZY, 051347Y, 051349Y,<br>05134AY, 05134JY, 05134KY,<br>05134ZY, 051407Y, 051409Y,<br>05140AY, 05140JY, 05140KY,<br>05140ZY, 051447Y, 051449Y,<br>05144AY, 05144JY, 05144KY,<br>05144ZY, 051507Y, 051509Y,<br>05150AY, 05150JY,05150KY,<br>05150ZY, 051547Y, 051549Y,<br>05154AY, 05154JY, 05154KY,<br>05154ZY, 051607Y, 051609Y,<br>05160AY, 05160JY, 05160KY,<br>05160ZY, 051647Y, 051649Y,<br>05164AY, 05164JY, 05164KY,<br>05164ZY, 059Y00Z, 059Y0ZZ,<br>059Y30Z, 059Y3ZZ, 05C00ZZ,<br>05C03ZZ, 05C04ZZ, 05C10ZZ,<br>05C13ZZ, 05C14ZZ, 05C30ZZ,<br>05C33ZZ, 05C34ZZ, 05C40ZZ,<br>05C43ZZ, 05C44ZZ, 05C50ZZ,<br>05C53ZZ, 05C54ZZ, 05C60ZZ,<br>05C63ZZ, 05C64ZZ, 05C70ZZ,<br>05C73ZZ, 05C74ZZ, 05C80ZZ,<br>05C83ZZ, 05C84ZZ, 05C90ZZ,<br>05C93ZZ, 05C94ZZ, 05CA0ZZ,<br>05CA3ZZ, 05CA4ZZ, 05CB0ZZ,<br>05CB3ZZ, 05CB4ZZ, 05CC0ZZ,<br>05CC3ZZ, 05CC4ZZ, 05CD0ZZ,<br>05CD3ZZ, 05CD4ZZ, 05CF0ZZ,<br>05CF3ZZ, 05CF4ZZ, 05CG0ZZ,<br>05CG3ZZ, 05CG4ZZ, 05CH0ZZ,<br>05CH3ZZ, 05CH4ZZ, 05CL0ZZ,<br>05CL4ZZ, 05CM0ZZ, 05CM3ZZ,<br>05CM4ZZ, 05CN0ZZ, 05CN3ZZ,<br>05CN4ZZ, 05CP0ZZ, 05CP3ZZ,<br>05CP4ZZ, 05CQ0ZZ, 05CQ3ZZ,<br>05CQ4ZZ, 05CR0ZZ, 05CR3ZZ,<br>05CR4ZZ, 05CS0ZZ, 05CS3ZZ,<br>05CS4ZZ, 05CT0ZZ, 05CT3ZZ,<br>05CT4ZZ, 05CV0ZZ, 05CV3ZZ,

		05CV4ZZ, 05CY0ZZ, 05CY3ZZ, 05CY4ZZ, 05HY02Z, 05HY32Z, 05HY42Z, 069300Z, 06930ZZ, 069330Z, 06933ZZ, 069340Z, 06934ZZ, 069Y00Z, 069Y0ZZ, 069Y30Z, 069Y3ZZ, 06C00ZZ, 06C03ZZ, 06C04ZZ, 06C10ZZ, 06C13ZZ, 06C14ZZ, 06C20ZZ, 06C23ZZ, 06C24ZZ, 06C30ZZ, 06C33ZZ, 06C34ZZ, 06C40ZZ, 06C43ZZ, 06C44ZZ, 06C50ZZ, 06C53ZZ, 06C54ZZ, 06C60ZZ, 06C63ZZ, 06C64ZZ, 06C70ZZ, 06C73ZZ, 06C74ZZ, 06C80ZZ, 06C83ZZ, 06C84ZZ, 06C90ZZ, 06C93ZZ, 06C94ZZ, 06CB0ZZ, 06CB3ZZ, 06CB4ZZ, 06CC0ZZ, 06CC3ZZ, 06CC4ZZ, 06CD0ZZ, 06CD3ZZ, 06CD4ZZ, 06CF0ZZ, 06CF3ZZ, 06CF4ZZ, 06CG0ZZ, 06CG3ZZ, 06CG4ZZ, 06CH0ZZ, 06CH3ZZ, 06CH4ZZ, 06CJ0ZZ, 06CJ3ZZ, 06CJ4ZZ, 06CM0ZZ, 06CM3ZZ, 06CM4ZZ, 06CN0ZZ, 06CN3ZZ, 06CN4ZZ, 06CP0ZZ, 06CP3ZZ, 06CP4ZZ, 06CQ0ZZ, 06CQ3ZZ, 06CQ4ZZ, 06CR0ZZ, 06CR3ZZ, 06CR4ZZ, 06CS0ZZ, 06CS3ZZ, 06CS4ZZ, 06CT0ZZ, 06CT3ZZ, 06CT4ZZ, 06CV0ZZ, 06CV3ZZ, 06CV4ZZ, 06CY0ZZ, 06CY3ZZ, 06CY4ZZ, 06HY02Z, 06HY32Z, 06HY42Z

eTable 3. Outcomes Stratified by Traumatic Brain Injury Severity

	**No CVD N (%)**	**Overall N (%)**	**CVD Death N (%)**	**Stroke N (%)**	**PAD N (%)**	**CAD N (%)**
No TBI	1,226,152 (97.41)	32,607 (2.59)	1466 (0.12)	7528 (0.60)	11,181 (0.89)	21,252 (1.69)
Mild TBI	242,272 (96.45)	8905 (3.55)	421 (0.17)	3293 (1.31)	2839 (1.13)	4964 (1.98)
Mod/Severe TBI	38,591 (94.17)	2387 (5.83)	99 (0.24)	1259 (3.07)	748 (1.83)	1011 (2.47)
Penetrating TBI	8150 (91.41)	864 (9.59)	14 (0.16)	546 (6.06)	286 (3.17)	330 (3.66)

All comparisons significant at p<0.001 level

© 2022 Stewart IJ et al. *JAMA Neurology.*

eTable 4. Weighted Baseline Characteristics of the Study Cohort After Inverse Propensity Score Weighting

	TBI History %	No TBI History %	Standardized Mean Difference†
Age at index date			
17-24	32.80	28.10	0.10
25-34	34.61	39.02	-0.09
35-44	17.91	18.74	-0.02
45-54	10.92	11.44	-0.02
55-64	3.01	2.46	0.03
≥ 65	0.74	0.24	0.07
Birth Year (median-IQR)	1981-14	1981-15	
Sex			-0.07
Male	78.97	81.73	
Female	21.03	18.27	
Race and Ethnicity			
Asian/Pacific Islander	6.03	6.15	-0.01
Hispanic	9.55	9.58	-0.001
Hispanic Black	0.57	0.54	0.005
Native American	1.70	1.72	-0.001
Non-Hispanic Black	17.35	17.23	0.003
Non-Hispanic White	62.79	63.07	-0.006
Unknown	2.02	1.70	0.02
Education			
Less than High School	1.41	1.42	-0.001
High School	65.40	67.12	-0.04
Some College	13.01	12.85	0.005
College Graduate	11.89	11.36	0.02
Graduate School	6.89	6.11	0.03
Unknown	1.40	1.13	0.02
Service Branch			
Army	43.67	45.40	-0.03
Air Force	21.91	19.68	0.05
Marines	13.73	14.98	-0.04
Navy/Coast Guard	20.58	19.83	0.02
Other	0.11	0.11	0.001
Component			
Guard	12.61	10.80	0.06
Reserve	37.55	37.94	-0.01
Active	50.04	51.26	-0.02
Rank			
Officer	13.96	12.59	0.04
Warrant	1.21	1.19	0.002
Enlisted	84.83	86.22	-0.04
Deployment History			
+Combat and Deploy	61.12	65.14	-0.08
+Combat/-Deploy	5.44	4.93	0.02
-Combat/+Deploy	5.19	5.05	0.01
-Combat or Deploy	28.25	24.87	0.08

© 2022 Stewart IJ et al. *JAMA Neurology.*

Smoking History	42.32	43.02	-0.01
Substance Use Disorder	11.49	11.47	0.001
Obesity	12.97	12.56	0.01
Obstructive Sleep Apnea	5.80	5.73	0.002
Insomnia	10.02	9.99	0.001
Post-Traumatic Stress Disorder	10.61	10.60	0.000
Depression	15.98	15.06	0.03
Anxiety	12.08	11.67	0.01
Hyperlipidemia	15.75	14.81	0.03
Kidney Disease	0.48	0.41	0.01
Hypertension	12.26	11.23	0.03
Diabetes	2.28	1.92	0.02

†Standardized mean difference for 2 groups is defined as the difference in IPS weighted means (for a continuous variable) or proportions (one category at a time for categorical variables) divided by pooled standard deviation. The overall standardized mean difference for a categorical variable with >2 categories can also be assessed by the normalized Mahalanobis distance based on weighted proportions (number of distinct categories minus one). We reported the former SMD for categorical variables for a more detailed evaluation. The stddiff SAS macro was used for the SMD calculation (https://support.sas.com/resources/papers/proceedings12/335-2012.pdf).

Imbalance between groups is defined as absolute value of SMD greater than 0.20 (Peter C. Austin. (2009) Balance diagnostics for comparing the distribution of baseline covariates between treatment groups in propensity-score matched samples. Statist. Med. 2009; 28:3083–3107).

eTable 5. Time-Varying Risk in Inverse Propensity Score–Weighted Models

**Time from**	**Mild TBI***			**Moderate/Severe TBI***			**Penetrating TBI***		
**Index**	**HR**	**95% CI**	**P**	**HR**	**95% CI**	**P**	**HR**	**95% CI**	**P**
3 Months	3.67	3.58-3.77	<0.001	7.51	7.33-7.69	<0.001	11.36	11.10-11.63	<0.001
6 Months	2.84	2.79-2.90	<0.001	5.09	5.00-5.19	<0.001	8.05	7.90-8.20	<0.001
1 Year	2.20	2.16-2.23	<0.001	3.45	3.40-3.50	<0.001	5.70	5.61-5.78	<0.001
2 Years	1.70	1.68-1.72	<0.001	2.34	2.31-2.37	<0.001	4.03	3.99-4.08	<0.001
3 Years	1.46	1.45-1.48	<0.001	1.87	1.84-1.89	<0.001	3.30	3.26-3.34	<0.001
4 Years	1.32	1.30-1.33	<0.001	1.59	1.57-1.61	<0.001	2.86	2.82-2.89	<0.001
5 Years	1.21	1.19-1.23	<0.001	1.40	1.38-1.42	<0.001	2.56	2.53-2.59	<0.001
6 Years	1.13	1.12-1.15	<0.001	1.27	1.25-1.28	<0.001	2.34	2.31-2.37	<0.001
7 Years	1.07	1.05-1.09	<0.001	1.16	1.14-1.18	<0.001	2.16	2.13-2.19	<0.001
8 Years	1.02	1.00-1.03	0.03	1.08	1.06-1.09	<0.001	2.02	1.99-2.05	<0.001
9 Years	0.97	0.96-0.99	0.002	1.01	0.99-1.02	0.28	1.91	1.88-1.94	<0.001
10 Years	0.94	0.92-0.95	<0.001	0.95	0.94-0.97	<0.001	1.81	1.78-1.84	<0.001

TBI: Traumatic brain injury HR: Hazard ratio CI: Confidence interval
*Compared to participants without TBI

© 2022 Stewart IJ et al. *JAMA Neurology.*
